# Bioassay-Guided Isolation and Identification of Xanthine Oxidase Inhibitory Constituents from the Fruits of *Chaenomeles speciosa* (Sweet) Nakai

**DOI:** 10.3390/molecules29184468

**Published:** 2024-09-20

**Authors:** Kui Li, Ruoling Xu, Mengting Kuang, Wei Ma, Ning Li

**Affiliations:** Anhui Key Laboratory of Bioactivity of Natural Products, School of Pharmacy, Anhui Medical University, Hefei 230032, China; lkui0912@foxmail.com (K.L.); xuruoling0126@gmail.com (R.X.); kuangmengting@ahmu.edu.cn (M.K.); mw421553449@sina.com (W.M.)

**Keywords:** *Chaenomeles speciosa* (Sweet) Nakai fruits, chemical composition, xanthine oxidase, uric acid, bioassay-guided, molecular docking

## Abstract

*Chaenomeles speciosa* (Sweet) Nakai (*C. speciosa*) is a traditional Chinese herbal medicine that possesses not only abundant nutritional value but also significant medicinal properties. The extracts of *C. speciosa* fruits effectively reduce urate levels, but the specific chemical constituents responsible for this effect in *C. speciosa* fruits are still unknown. Therefore, this study aims to investigate and analyze the structure–activity relationships of these constituents to better understand their ability to lower uric acid. Activity-guided fractionation and purification processes were used to isolate compounds with xanthine oxidase (XO) inhibitory activity from *C. speciosa* fruits, resulting in three extracts: petroleum ether, ethyl acetate, and *n*-butanol. The ethyl acetate and *n*-butanol fractions showed strong activity and underwent further separation and purification using chromatographic techniques. Twenty-four compounds were isolated and identified, with nine showing potent activity, including chlorogenic acid, methyl chlorogenate, butyl chlorogenate, ethyl chlorogenate, cryptochlorogenic acid methyl ester, caffeic acid, *p*-coumaric acid, benzoic acid and protocatechuic acid. The docking analysis showed that these compounds interacted with amino acid residues in the active site of XO through hydrogen bonding and hydrophobic interactions. These findings suggest that these compounds help reduce uric acid in *C. speciosa*, supporting further investigation into their mechanism of action.

## 1. Introduction

Xanthine oxidase (XO) is an enzyme that catalyzes hypoxanthine to produce xanthine, which is further converted into uric acid. Additionally, XO can directly convert xanthine into uric acid while releasing superoxide [1,2], and uric acid, as the end product of purine metabolism in the human body, which accumulates in blood, can lead to hyperuricemia [3]. The molybdopterin (Mo-pt) domain of XO is an important catalytic center, where xanthine oxidation occurs [4]. Due to its significant regulatory effect on uric acid production, inhibiting XO activity has become an effective approach for controlling both uric acid generation and free radical release, thereby treating hyperuricemia [5]. Hence, it is important to investigate XO inhibitors that are effective in preventing hyperuricemia.

XO inhibitors have been predominantly employed as an anti-hyperuricemia therapy for gout treatment [6]. The main drugs used clinically for hyperuricemia treatment include allopurinol (AP), febuxostat, and other medications. However, these drugs are associated with notable side effects. Allopurinol has been reported to frequently cause renal damage [7], while febuxostat often leads to adverse cardiovascular events [8]. Therefore, it is imperative to identify drugs that can effectively treat hyperuricemia without toxic side effects. Chinese herbal medicines (CHMs) represent evidence-based therapies with desirable efficacy and acceptable safety profiles, thus serving as valuable sources for discovering safe agents that lower serum urate levels in the management of hyperuricemia-related diseases [7].

The plant *Chaenomeles speciosa* (Sweet) Nakai (*C. speciosa*), which belongs to the genus *Chaenomeles* in the Rosaceae family, is a native temperate plant extensively cultivated in Asia and Europe. It has been widely utilized in the medicine and functional food industries [9]. Pharmacological investigations have revealed that this plant exhibits a diverse range of attributes, including anti-inflammatory and analgesic activities, antioxidant activities, antihyperglycemic and antihyperlipidemic activities, gastrointestinal protective activities, as well as antitumor and immunomodulatory activities [10]. Our research group has previously conducted a study on the urate-lowering effect of *C. speciosa* [11], and it has been confirmed that the *C. speciosa* extract exhibited significant inhibitory effects on XO and demonstrated promising therapeutic potential for hyperuricemia in rats. However, further exploration is required to elucidate the specific components of *C. speciosa* extract and their underlying molecular mechanisms. Therefore, in this study, we further revealed the active parts and components of *C. speciosa* with urate-lowering effects, and analyzed the material basis of its therapeutic effect.

## 2. Results

### 2.1. In Vitro Inhibition of XO by AP and Three Extracts of C. speciosa

**Figure 1:**
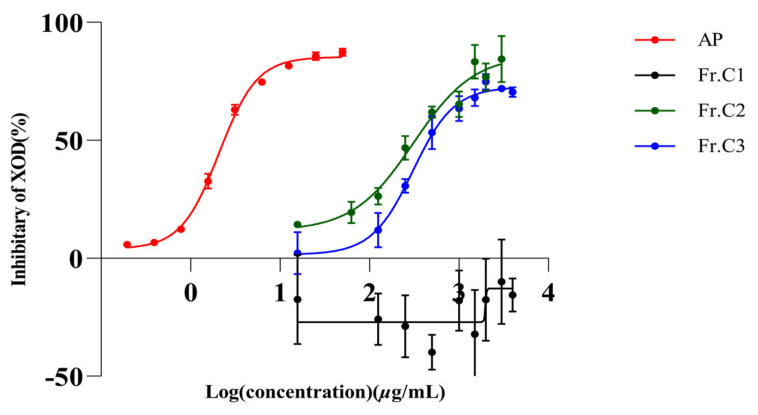
Four *C. speciosa* fruit fractions, petroleum ether fraction (Fr.C1), ethyl acetate fraction (Fr.C2) and *n*-butanol fraction (Fr.C3), and AP inhibited XO in vitro. Values are expressed as means = SD.

The inhibitory effects of the petroleum ether fraction (Fr.C1), ethyl acetate fraction (Fr.C2), *n*-butanol fraction (Fr.C3) and AP on XO activity are illustrated in Figure 1. Among these fractions, only Fr.C1 did not exhibit any inhibition towards XO, while the remaining two fractions demonstrated dose-dependent inhibition towards XO. The IC_50_ values for Fr.C2, Fr.C3, and AP were determined to be 341.80 µg/mL, 321.10 µg/mL and 2.08 µg/mL (Table 1), respectively. The findings indicate that ethyl acetate fraction and *n*-butanol fraction exhibited superior inhibitory effects on XO in comparison to the petroleum ether fraction.

### 2.2. Monomer Compounds of C. speciosa and Its Inhibitory Effect on XO

**Figure 2:**
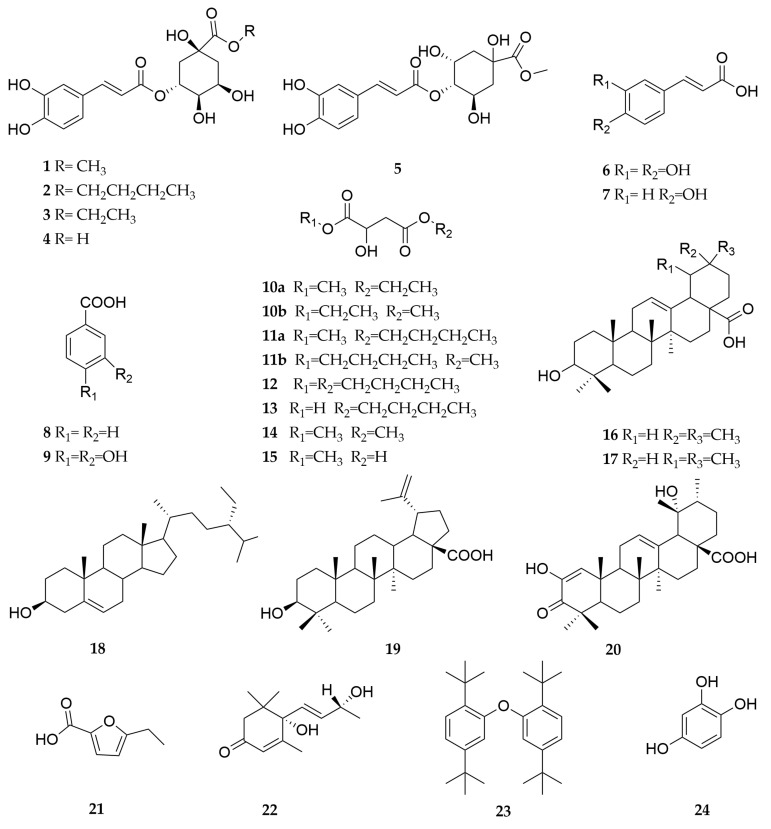
Structure of compounds **1**–**24** isolated from *C. speciosa*.

The known compounds (**1**–**24**) were identified by comparing their experimental NMR spectral data with the corresponding data reported in the literature. These compounds were characterized as methyl chlorogenate (**1**) [12], butyl chlorogenate (**2**) [13], ethyl chlorogenate (**3**) [14], chlorogenic acid (**4**) [15], cryptochlorogenic acid methyl ester (**5**) [14], caffeic acid (**6**) [16], *p*-coumaric acid (**7**) [17], benzoic acid (**8**) [18], protocatechuic acid (**9**) [19], ethylmethyl malate isomers (**10a**/**10b**) [20], butylmethyl malate isomers (**11a**/**11b**) [21], bibutyl malate (**12**) [22], butyl 2-hydroxysuccinate (**13**) [23], dimethylmalate (**14**) [24], methylmalate (**15**) [25], oleanolic acid (**16**) [26], ursolic acid (**17**) [27], *β*-sitosterol (**18**) [28], betulinic acid (**19**) [29], fupenzic acid (**20**) [30], 5-hydroxymethylfurfural (**21**) [31], vomifoliol (**22**) [32], 2, 2′-oxybis (1, 4-di-tert-butylbenzen) (**23**) [33], and 1, 2, 4-benzenetriol (**24**) [34] (Figure 2). It is noteworthy that compounds **1**–**15**, **18**–**24** were isolated and identified from *C. speciosa* for the first time.

### 2.3. Inhibition of XO by Monomeric Compounds in C. speciosa

**Figure 3:**
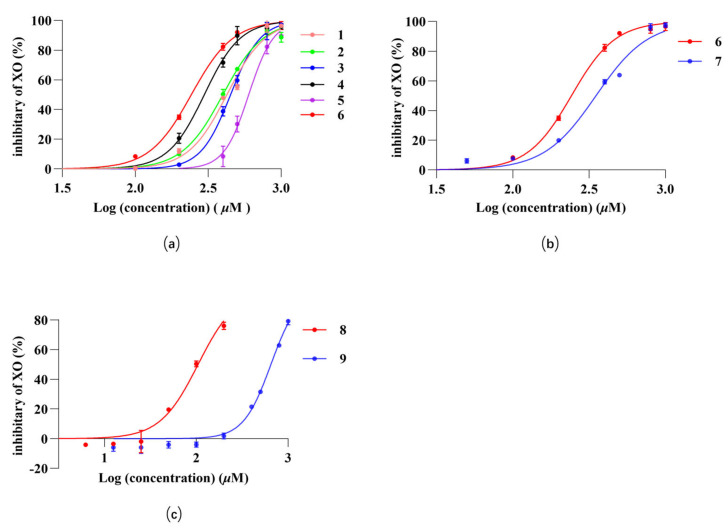
(**a**–**c**) Inhibition of XO by compounds **1**–**9** in vitro. Values are expressed as means = SD.

Table 2 showed the concentrations at which compounds **1**–**24** inhibited half of the XO activity, and the values are expressed as IC_50_. Figure 3 illustrated the inhibitory effects of compounds **1**–**9** on XO activity. Figure 3a showed that compounds **1**–**6** exhibited inhibitory activity against XO. Notably, these compounds contained a coffee acyl group, and the data indicated a dose-dependent inhibition of XO by this compound class. When the concentration was lower than 200 µM, there was basically no inhibition, and when the concentration was around 1000 µM, the inhibition rate reached almost 100%. Compared to compound **6**, there was not a significant difference in the XO inhibiting effect of compounds **1**–**5**. Therefore, we supposed that the decisive role in coffee’s acyl group lies in the chlorogenic acid ester compounds’ rate of XO inhibition. Figure 3b illustrated the in vitro inhibition of XO by compounds **6** and **7**. At the same concentration, the inhibition rate of compound **6** on XO was higher than that of compound **7**. An analysis of its molecular structure revealed that compound **6** had one more phenolic hydroxyl group than compound **7**. Therefore, we speculated that the hydroxyl group on the benzene ring of this class of compounds could increase the inhibition of XO. Figure 3c illustrated the inhibition of XXO by benzoic acid compounds. These compounds also inhibited XO in a dose-dependent manner, but compound **8** showed a high inhibition at a concentration of less than 200 µM, while compound **9** achieved the same inhibition at a concentration of about 1000 µM. An observation of its molecular structure revealed that compound **9** had two more phenolic hydroxyl groups than compound **8**. Thus, we speculated that replacing benzoic acid compounds’ hydroxyl groups on the benzene ring will lower its inhibitory effect on XO.

### 2.4. Molecular Docking Analysis

**Figure 4:**
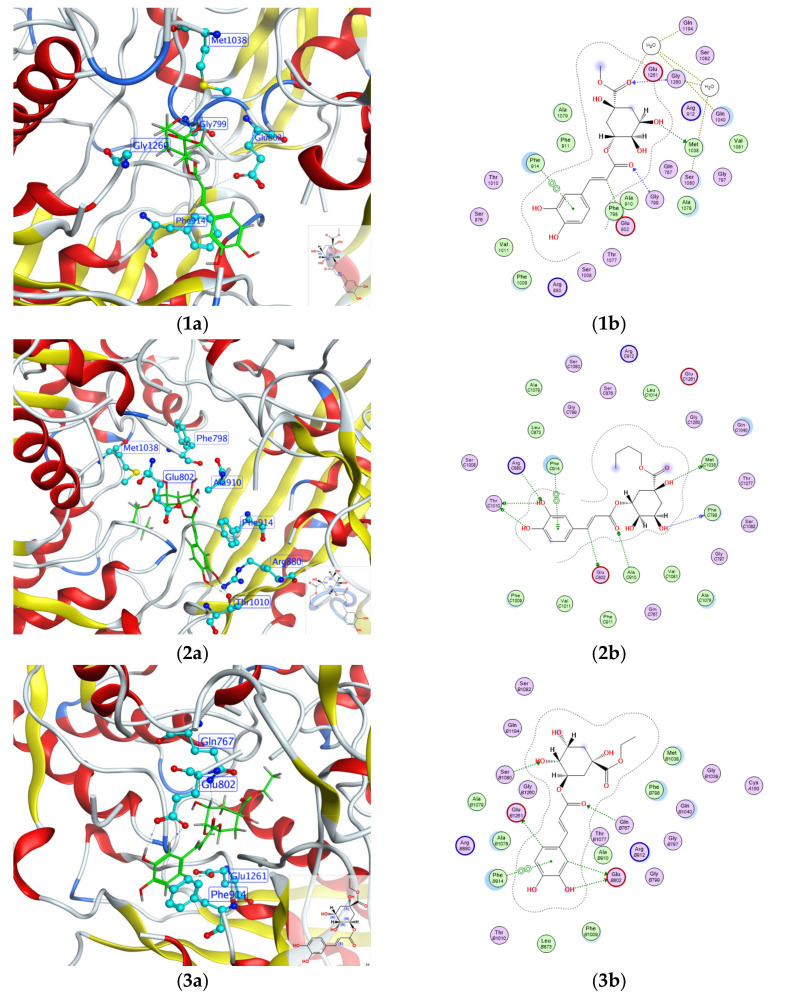

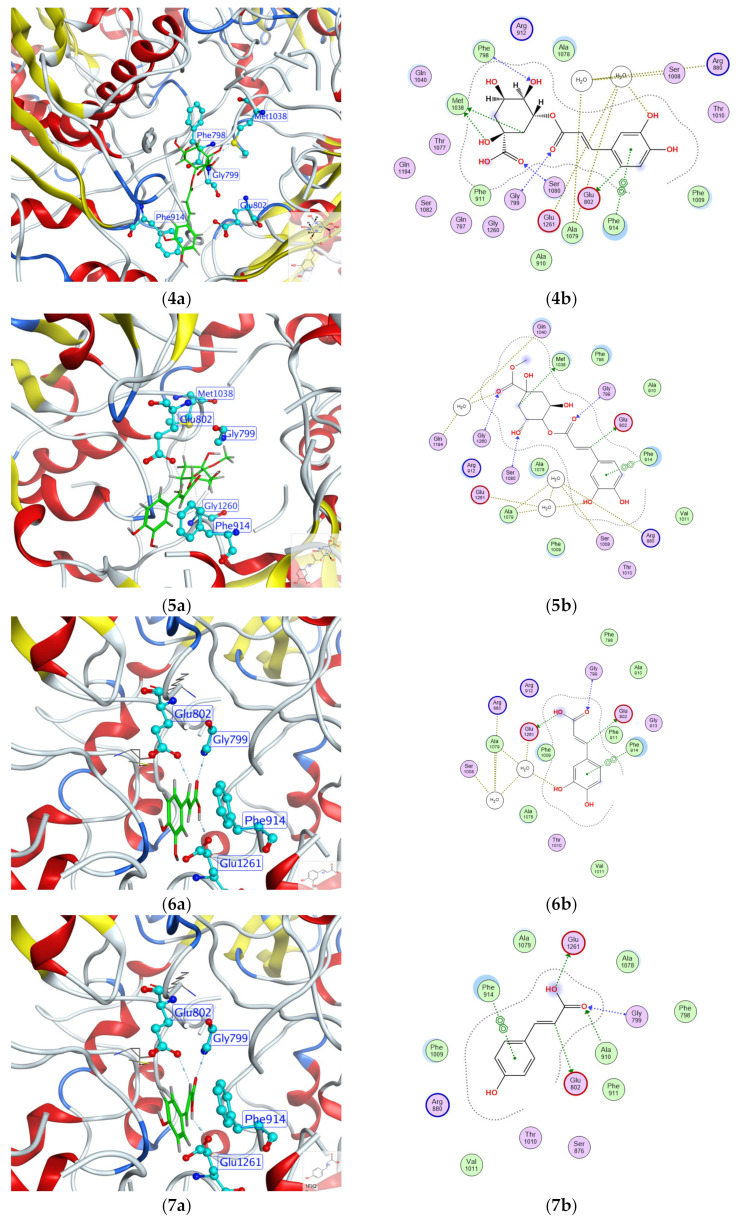

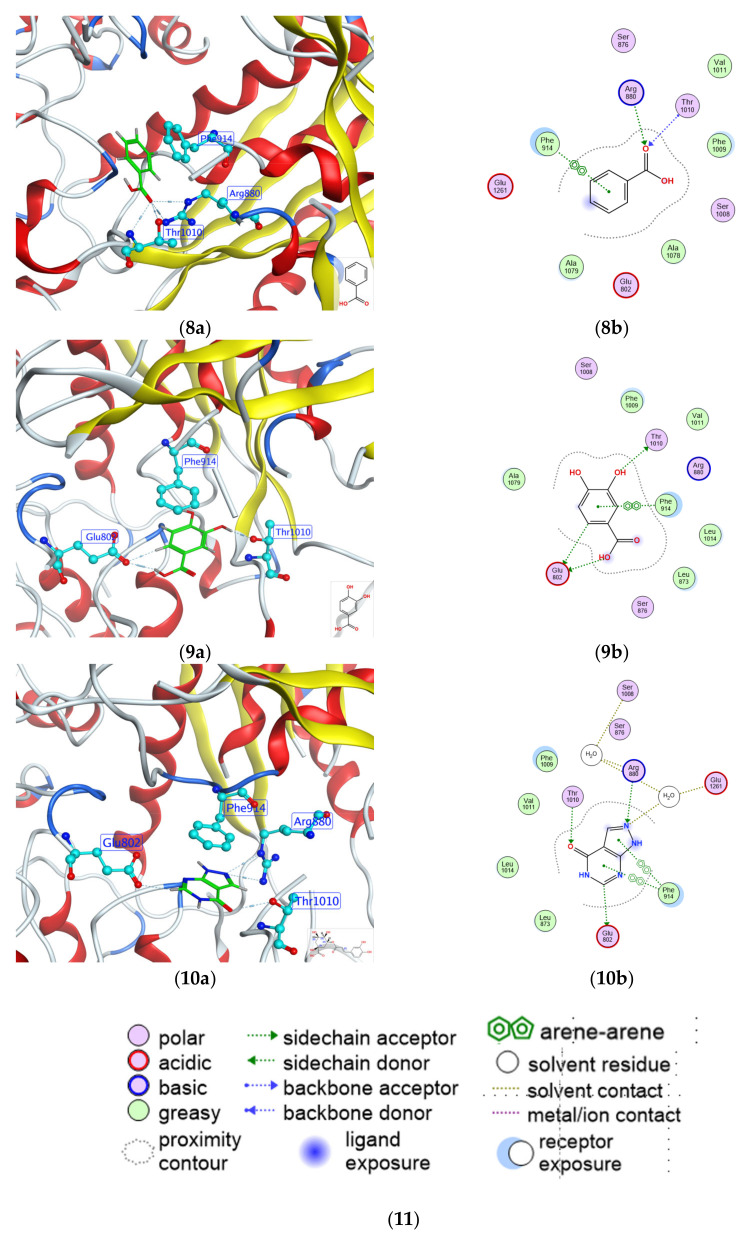
Molecular docking images of compounds **1**–**9** and AP with XO. (**1a**–**10a**) The binding orientation of compounds **1**–**9** and AP inside the binding site of XO; (**1b**–**10b**) The 2D ligand–protein interaction of compounds **1**–**9** and AP with residues of XO. (**11**) Legend for the 2D images.

Molecular docking has been frequently utilized for the analysis of ligand–protein binding modes. In this study, we focused on the highest inhibition of XO and conducted a conformational analysis of molecular docking to determine the binding constants. Figure 4 illustrates the docking results of compounds **1**–**9** with XO (PDBID: 1FIQ). The molybdopterin (Mo-pt) domain is the functional site of XO in which the oxidation of xanthine to uric acid occurs; Arg880, Phe1009, Phe914, Glu802, Asn768, Thr1010, Val1011, Leu873, and Glu1216 are its critical amino acids [35]. Compound **1** showed interaction with Glu802, Met1038, Gly799, Gly1260 and Phe914 of the XO 1FIQ protein, with bonding distances of 3.48 Å, 3.28 Å, 3.12 Å, 3.40 Å, 3.37 Å, respectively. Compound **2** exhibited interactions with Thr1010, Glu802, Met1038, Phe798, Arg880, Ala910, and Phe914, with bonding distances of 3.09 Å, 3.41 Å, 3.31 Å, 2.90 Å, 3.34 Å, 3.30 Å, and 3.63 Å, respectively. Compound **3** demonstrated interaction with Glu1261, Glu802, Gln767, and Phe914, with bonding distances of 3.33 Å, 3.43 Å, 3.28 Å, and 3.43 Å, respectively. Compound **4** displayed interactions with Glu802, Met1038, Gly799, Phe798, and Phe914, with bonding distances of 3.31 Å, 3.05 Å, 3.07 Å, 3.31 Å, and 3.30 Å, respectively. Compound **5** showed interactions with Glu802, Met1038, Gly799, Gly1260, and Phe914, with bonding distances of 3.35 Å, 3.89 Å, 3.39 Å, 3.48 Å, and 3.30 Å, respectively. Compound **6** exhibited interactions with Glu802, Glu1261, Gly799, and Phe914, with bonding distances of 3.23 Å, 2.71 Å, 3.17 Å, and 3.39 Å, respectively. Compound **7** demonstrated interactions with Glu802, Glu1261, Gly799, and Phe914, with bonding distances of 3.28 Å, 2.71 Å, 3.46 Å, and 3.46 Å, respectively. Compound **8** displayed interactions with Arg880, Thr1010, and Phe914, with bonding distances of 3.13 Å, 3.03 Å, and 3.46 Å, respectively. Compound **9** showed interactions with Glu802, Thr1010, and Phe914, with bonding distances of 3.14 Å, 2.88 Å, and 3.61 Å, respectively. AP showed interactions with Glu802, Arg880, Thr1010, and Phe914, with bonding distances of 3.21 Å, 3.11 Å, and 2.97 Å, respectively. The binding energies for compound **1**–**9** were found to be −8.77, −7.76, −8.67, −8.45, −9.21, −5.90, −5.68, −5.08, −5.44 kcal/mol, respectively. The binding energy of ap to XO was −4.18 kcal/mol. The results demonstrated that compounds **1**–**9** and AP effectively occupied the functional site of XO and interacted with the key amino acids in the Mo-pt domain, and compounds **1**–**9** had a good binding affinity to XO compared with the AP. This indicates that the ligand molecules exhibit a greater affinity for the protein active site and therefore hold a promising place as effective inhibitors for XO.

## 3. Discussion

XO is one form of xanthine oxidoreductase (XOR), with a molecular weight of about 300 kd. Native XO contains two subunits with a molecular weight of approximately 150 kd [1], and its molybdopterin (Mo-Pt) domain is an important catalytic center when xanthine and hypoxanthine are oxidated [36]. XO catalyzes the oxidative hydroxylation of hypoxanthine to xanthine and xanthine to uric acid, the final two steps in purine catabolism in humans [37]. Uric acid deposition directly triggers the development of gout, which has been increasingly prevalent over recent years. The prevalence of hyperuricemia is approximately 20% in America and even higher in Korea, reaching up to 26.6% [38]. In China, with a quarter of the world’s population, the incidence of gout stands at about 16.6% [39] Hence, it is imperative to discover XO inhibitors in order to suppress the synthesis of uric acid and decrease the prevalence of hyperuricemia.

Currently, XO inhibitors such as allopurinol and febuxostat are primarily employed for the clinical management of hyperuricemia [40]. However, these medications have been associated with cardiovascular diseases, including heart failure, ischemic heart disease, hypertension, and cardiomyopathy [41]. Therefore, there is an urgent need to find XO inhibitors with fewer side effects for the treatment of hyperuricemia.

Thousands of years ago, extensive research was conducted on the use of food and herb medicines for treating gout and hyperuricemia. *Cordyceps militaris*, *Poria cocos*, and *G. applanatum* [42] were among the examples studied. Based on our previous reports [11], *C. speciosa*, a traditional Chinese medicinal material used as both medicine and food with minimal side effects, has been confirmed to possess uric acid-lowering properties. Therefore, we believe that the active components can be extracted and isolated from *C. speciosa*, which may have the effect of reducing uric acid, and provide some theoretical basis for the development of safe and effective drugs for the treatment of hyperuricemia.

This study aims to identify chemical compositions from *C. speciosa* fruits that can effectively inhibit XO activity by conducting in vitro enzyme activity screening on extracts obtained from this plant material. It was observed that the petroleum ether fraction had no inhibition of XO, while the ethyl acetate fraction and *n*-butanol fraction exhibited significant inhibitory effects on XO (Figure 1); Therefore, we focused primarily on isolating small molecules from the ethyl acetate and *n*-butanol fractions. A total of 24 compounds were isolated from these two fractions, and subsequent in vitro enzyme activity tests revealed that compounds **1**–**9** all demonstrated inhibitory effects on XO activity. The compounds **1**–**9** were structurally identified as chlorogenic acid and its esters, caffeic acid, and benzoic acid derivatives. Henceforth, it is reasonable to assume that chlorogenic esters, caffeic acids, and benzoic acids are the main urate-lowering chemicals present in *C. speciosa*. This study demonstrated that **1**–**6** (Figure 3a) exhibited a dose-dependent effect on XO, effectively inhibiting XO activity at concentrations up to 1000 µM. Furthermore, altering the quininate ester groups did not significantly impact its inhibitory effect on XO. Hence, this study suggests that caffeic acid group plays a pivotal role in XO inhibition. Figure 3b compares the effect of caffeic acid on XO with p-coumaric acid (**7**), which lacks a hydroxyl group on a benzene ring compared to caffeic acid, and the inhibition results show that caffeic acid exhibits better inhibition at low concentrations. Therefore, we suggest that the hydroxyl group on the benzene ring of these compounds inhibits XO. Noriyoshi Masuoka and Isao Kubo [43] suggested that XO inhibitors should possess alkyl chains and specific hydroxy group arrangements in the phenol portion. Yuan-Ching Chang et al. [44] proposed that C6-C3 phenylpropanoids are effective XO inhibitors, particularly caffeic acid and caffeic acid phenethyl ester, which exhibit significant XO inhibitory activity. They also indicated that the hydroxyl arrangement on the benzene ring and carboxyl esterification on the long chain greatly influence XO activity. Urszula Gawlik-Dziki et al. [45] discovered the noncompetitive inhibition of XO by chlorogenic acid. Yin Wan et al. [3] found that dicaffeoylquinic acids (diCQAs) have a stronger XO inhibitory effect compared to monocaffeoylquinic acids (monoCQAs). It is worth noting that **1**–**5** are all structures esterified with both caffeic acid groups and quininate ester groups, both of which have been reported to possess XO inhibitory activity. Among them, caffeic acid primarily exerts competitive inhibition on XO, while chlorogenic acid exhibits a non-competitive inhibitory effect on XO [46]. These views support the accuracy of the experimental results. Figure 3c compares the inhibition of XO by benzoic acid and protocatechuic acid, and the results show that the inhibition of benzoic acid is better than that of protocatechuic acid at low concentrations. By analyzing the structure of this class of compounds, we found that compound **9** has two more phenolic hydroxyl groups than compound **8.** Therefore, we thought that the hydroxyl group on the benzene ring of this class of compounds would reduce the inhibition of XO. Falodun et al. [47] reported the XO inhibitory properties of benzoic acid (**8**), while Jun Li et al. [48] found protocatechuic acid (**9**) to possess similar effects on XO inhibition. Another investigation [42] revealed that 2, 4-dihydroxybenzoic acid methyl ester (DA) also exhibits an inhibitory effect on XO, but its inhibition rate at the same concentration is lower than that of protocatechuic acid. These experimental results also confirmed the correctness of our experimental results. Studies have shown that phenolic acids have antioxidant activity [49], and it has been reported that chlorogenic acid (**4**), caffeic acid (**6**), *p*-coumaric acid (**7**), and protocatechuic acid (**9**) have high antioxidant activity [50,51]. In addition, XO catalyzes the production of hypoxanthine from xanthine mainly at Mo-pt domain, which in turn catalyzes the production of uric acid from hypoxanthine. Therefore, we hypothesized that phenolic acids such as compounds **4**, **6**, **7**, **9** might exert their urate-lowering effects by inhibiting the REDOX reaction of XO at the active site.

To gain insights into the atomic-level binding modes of **1**–**9**, their 3D structures were simulated using Molecular Operating Environment (MOE) software and subsequently subjected to molecular docking studies with XO (PDB ID:1FIQ) [52]. The binding energies for compound **1**–**9** were found to be −8.77, −7.76, −8.67, −8.45, −9.21, −5.90, −5.68, −5.08, −5.44 kcal/mol, respectively. The present study showed that compounds **1**–**9** displayed good interaction with the amino acid residues at the active site, as indicated by their binding energies. In addition, it was also shown that compounds **1**–**9** could interact with key amino acid residues in the Mo-pt domain, such as Phe914 and Glu802. It can be concluded that compounds **1**–**9** can act in the active center of XO and inhibit XO activity through hydrogen bonding and π-π conjugation.

Taken together, it is reasonable to suggest that chlorogenic acid and its esters, caffeic acid, and benzoic acid compounds are the main urate-lowering chemicals in *C. speciosa* fruits. These compounds could be used as potential drugs for the prevention and treatment of hyperuricemia in clinic.

## 4. Materials and Methods

### 4.1. Instrumentation and General Experimental Techniques

The 1D and 2D NMR spectra were recorded on an Avance III-600 spectrometer (Bruker, Billerica, MA, USA), with TMS serving as an internal standard. Chemical shifts (*δ*) are expressed in ppm. Sephadex LH-20 gel (25–100 µm) was sourced from Pharmacia Fine Chemical located in Uppsala, Sweden. C18 silica gel (50 µm) was obtained from YMC (Kyoto, Japan). Silica gel 60 F254 aluminum sheets (Merck, Darmstadt, Germany) were utilized for thin-layer chromatography (TLC). Spots on the TLC plate were detected by being exposed to UV light (254 nm) and by spraying with p-anisaldehyde reagent. Column chromatography silica gel (200–300 mesh) was purchased from Shanghai Haohong Biomedical Technology in Shanghai, China. HPLC separations were performed using a Thermo Scientific UltiMate3000 liquid chromatography system (Waltham, MA, USA). A Thermo Hypersil ODS column (ODS, 250 × 10 mm, 5 µm; Thermo, Waltham, MA, USA) was employed. Xanthine (X7375), XO (4376-5UN), and AP (A8003) were supplied by Sigma-Aldrich LLC, a company based in St. Louis, MO, USA. MOE 2012 software was acquired by the Department of Medicinal Chemistry at Anhui Medical University (Hefei, China).

### 4.2. Plant Materials

The fruits of *C. speciosa* were furnished by Anhui Xiehecheng Chinese Herb Limited Corporation based in Bozhou, China in September 2021. Prof. Kai-Jin Wang of Anhui University provided authentication. A voucher specimen (20210930) was placed at the School of Pharmacy, Anhui Medical University.

### 4.3. Extraction, Isolation and Structure Identification

The plant materials of *C. speciosa* fruits (10 kg) were successively extracted in 95%, 85%, and 75% ethanol (at a ratio of 1:10 *w*/*v*) at a temperature of 60 °C for 3 h. The resulting filtrate was collected and subsequently mixed before being concentrated using a rotary evaporator (operating at a temperature of 60 °C and a speed of 45 rpm), yielding approximately 2904 g of crude residue. This residue was then resuspended in water and subjected to extraction with petroleum ether, ethyl acetate, and *n*-butyl alcohol to obtain three fractions: petroleum ether fraction (Fr.C1, 132.3 g), ethyl acetate fraction (Fr.C2, 532.5 g) and *n*-butanol fraction (Fr.C3, 926.2 g).

### 4.4. Inhibitory Assays of XO by Different Extracts of C. speciosa Fruits In Vitro

According to the previously determined method, the optical lighting method is measured by measuring the generation of uric acid of the elasticine [11]. Mixed solutions are prepared in the phosphate buffer (0.01 M, pH 7.5), which contains XO (0.01 units/mL of constant concentration; 1 unit converts 1.0 µmol xanthine to 25 °C and PH 7.5). Add the different concentrations of each extract of *C. speciosa* fruits and incubate for 15 min at 25 °C. Start the response by adding a lot of substrates (9 mM), and the reaction is terminated after 15 min of incubation by adding hydrochloric acid (50 µL, concentration: 1 N). Vortex and centrifuge the hybrid solution, and use the upper liquid to measure the value of the optical light at 290 nm. A positive drug control (AP) was included for comparative analysis. The inhibition ratio of XO was calculated by using the following formula:

$$Inhibition\;ratio\left(\%\right)=\left(1-\frac{S-S_{0}}{B-B_{0}}\right)\times 100$$

The experiments performed on the sample in the presence and absence of XO are, respectively, labeled as S and S_0_. The enzyme activity when there is no sample is denoted as B, and B_0_ represents the control of B when neither the sample nor XO is present.

### 4.5. Extraction and Separation of Monomer Compound

The ethyl acetate extract (Fr.C2, 532.5 g) was subjected to column chromatography on silica gel (200–300 mesh) using gradients of petroleum ether (PE)/CH_2_Cl_2_ (from 1:1 to 0:1, *v*/*v*) and CH_2_Cl_2_/MeOH (from 100:1 to 0:1, *v*/*v*), resulting in the isolation of seven fractions (Fr.C2.1–Fr.C2.7), which were monitored by thin-layer chromatography. Nine sub-fractions (Fr.C2.2.1–Fr.C2.2.9) were obtained from the partition of Fr.C2.2 (41.9 g) through silica gel column chromatography, eluted with a CH_2_Cl_2_/MeOH gradient ranging from 100:1 to 1:1. The sample Fr.C2.2.8 (26.2 g) was fractionated using a Sephadex LH-20 column, eluted with a gradient of MeOH/H_2_O (ranging from 10:90 to 100:0), resulting in the isolation of four subfractions (Fr.C2.2.8.1–Fr.C2.2.8.4). Compounds **10a**/**10b** (6732 mg) and **15** (700 mg) and were obtained from Fr.C2.2.8.3 through silica gel column chromatography, using CH_2_Cl_2_/MeOH as the eluent in a ratio of 100:1. Fr.C2.9 (99 g) was separated by silica gel column chromatography (200–300 mesh), employing CH_2_Cl_2_/MeOH gradients ranging from 100:1 to 0:1, *v*/*v*, yielding nine fractions (Fr.C2.9.1–Fr.C2.9.9), which were monitored by TLC. Compound **16** (88 mg) was isolated from FrC2.9.3 via silica gel column chromatography using CH_2_Cl_2_/MeOH as the eluent in a ratio of 40:1. FrC2.9.4 underwent purification on a Sephadex LH-20 column using MeOH as the solvent and further purified through preparative TLC, utilizing CH_2_Cl_2_/MeOH at a ratio of 60:1, leading to the isolation of **17** (20 mg). Fr.C2.5 (101.2 g) was subjected to MCI column elution with MeOH/H_2_O (from 10:90 to 100:0) to yield five subfractions (Fr.C2.5.1 to Fr.C2.5.5). Fr.C2.5.1 (53.1 g) was separated on a Sephadex LH-20 column eluted with MeOH/H_2_O (from 10% to 100%) and purified by preparative TLC (CH_2_Cl_2_/MeOH, 40:1) to afford **14** (13.1 mg). Fr.C2.5.2 (9.1 g) was separated by an Sephadex LH-20, eluted with isocratic of MeOH/H_2_O (from 10% to 100%, *v*/*v*) to afford 8 fractions (Fr.C2.5.2.1–Fr.C2.5.2.8) monitored by TLC. Fr.C2.5.2.1 (2.0 g) was separated by Sephadex LH-20, column eluted with MeOH/H_2_O (from 10% to 100%) and purified by silica gel CC with CH_2_Cl_2_/MeOH (30:1) to produce **11a**/**11b** (273.2 mg), **12** (17.1 mg), **13** (219.3 mg). Fr.C2.5.2.4 was separated by Sephadex LH-20 column, eluted with MeOH/H_2_O (from 10% to 100%) to yield **21** (62 mg). Fr.C2.5.2.5 was isolated by Sephadex LH-20, eluted with MeOH/H_2_O (from 10% to 100%) and purified by silica gel CC, eluted with CH_2_Cl_2_/MeOH (30:1) to afford **22** (16 mg). Fr.C2.5.3 was subjected to a Sephadex LH-20 column (MeOH) and purified by preparative TLC (CH_2_Cl_2_/MeOH, 50:1) to yield **18** (7 mg), **19** (114 mg) and **23** (139 mg). The *n*-BuOH extract (Fr.C3, 926.6 g) was separated to silica gel (200–300 mesh) CC with CH_2_Cl_2_/MeOH gradients (from 100:1 to 0:1, *v*/*v*) to afford 6 fractions (Fr.C3.1–Fr.C3.6). Seven fractions (Fr.C3.4.1–Fr.C3.4.7) were obtained from the Fr.C3.4 (384.6 g) partition by silica gel CC, eluted with CH_2_Cl_2_/MeOH (from 40:1 to 1:1). Compounds **6** (18 mg) and **7** (9 mg) were isolated from the Fr.C3.4.3 partition by silica gel column chromatography and eluted with CH_2_Cl_2_/MeOH (30:1). Fr.C3.4.4 was isolated by C18, eluted with MeOH/H_2_O (from 10% to 100%) and purified by HPLC (45%, MeOH/H_2_O) to afford **8** (27 mg) and **9** (20 mg). Compound **20** (38 mg) was isolated from the Fr.C3.4.5 partition by silica gel column chromatography and eluted with CH_2_Cl_2_/MeOH (40:1). Fr.C3.4.6 (40 g) was separated by an Sephadex LH-20 eluted with isocratic of MeOH/H_2_O (from 10% to 100%, *v*/*v*) to yield **2** (75 mg) and **24** (28 mg). Fr.C3.4.6 was isolated by ODS, eluted with MeOH/H_2_O (from 10% to 100%) to afford **1** (12 mg) and **4** (20 mg). Fr.C3.4.7 was purified using semipreparative HPLC (MeOH/H_2_O, 60:40, *v*/*v*, 2.5 mL/min) to yield **5** (6 mg) and **3** (3 mg).

### 4.6. Molecular Docking

To ensure the accuracy of our target selection, we considered several criteria to guarantee the precision of our results. First, we limited our selection to PDB structures that contained a predefined ligand which had been reported before. This is of great importance as it makes sure that our results can be compared to previous studies and are consistent with the existing literature. Second, the docking protocol was validated by docking with the internal ligands extracted from the obtained PDB IDs of the target candidates. The protocol with the best docking pose of the internal ligand, as reported in the respective PDB, was chosen. The crystal structures of the candidate protein targets for XO were retrieved from the RCSB Protein Data Bank (http://www.pdb.org/, PDBID: 1FIQ;) [52]. There was no solvent present, and all heteroatoms and water molecules were eliminated.

The structures of compounds **1**–**9** and AP were drawn and then saved in sdf format and imported into the MOE software database. The structures of compounds **1**–**9** and AP were washed to obtain their 3D conformation using the wash function under the computer panel. Amino acids (Arg880, Phe1009, Phe914, Glu802, Asn768, Thr1010, Val1011, Leu873, and Glu1216) in the active central site (Mo-pt domain) were selected in the target protein (1FIQ), and docking targets were created using the set create function under the select panel. The interactions between XO inhibitors and target candidates were assessed by docking which was carried out by utilizing selected parameters (Placement: Triangle Matcher, Refinement: Rigid Receptor, Poses: 30). Finally, the S score was employed as a basis to select the top five targets, calculated by the default scoring built-in function in MOE is the S value, which is a scoring number that evaluates the affinity of the ligand with the receptor [53]. For further analysis, 2D protein–ligand interaction images were obtained using the ligand interactions option in MOE. The energies of docking complexes and H-bonding length were incorporated in the ligand interaction analysis. Then, the best five targets were imported into the STRING 12.0 database and analyzed for co-expression.

## 5. Conclusions

In this study, 24 compounds were isolated from the active ethyl acetate and *n*-butanol fractions of *C. speciosa* fruits extract, and their XO inhibitory activity was assessed by measurement. Among these compounds, methyl chlorogenate, butyl chlorogenate, ethyl chlorogenate, chlorogenic acid, cryptochlorogenic acid methyl ester, caffeic acid, p-coumaric acid, benzoic acid and protocatechuic acid exhibited superior XO inhibitory effects. The molecular docking analysis suggested that **1**–**9** inhibit XO activity by binding to the active catalytic site, thereby preventing substrate entry and inducing conformational changes in XO. However, the specific binding sites between the protein and xo remain unclear and require further investigation. Overall, this study investigated the active substances in *C. speciosa* responsible for reducing uric acid levels, providing a theoretical foundation for drug development and functional food products aimed at preventing hyperuricemia.

## Figures and Tables

**Table 1:** Inhibitory effects of *C. speciosa* fruit extracts and AP on XO in vitro.

Extract	IC_50_ (µg/mL)
Fr.C1	nc
Fr.C2	341.80
Fr.C3	321.10
AP	2.08

nc: not calculated. IC_50_: inhibition of XO 50% activity (concentration in µg/mL required for a 50% reduction in XO activity).

**Table 2:** Inhibitory effects of compounds (**1**–**9**) on XO in vitro.

NO.	Compound	Classification	IC_50_ (µg/mL)
1	methyl chlorogenate	penylpropanoids	156.5
2	butyl chlorogenate	penylpropanoids	163.6
3	ethyl chlorogenate	penylpropanoids	172.2
4	chlorogenic acid	penylpropanoids	105.4
5	cryptochlorogenic acid methyl ester	penylpropanoids	220.9
6	caffeic acid	hydroxycinnamic acid	43.6
7	p-coumaric acid	hydroxycinnamic acid	57.6
8	benzoic acid	aromatic carboxylic acids	12.7
9	protocatechuic acid	aromatic carboxylic acids	100.27

IC_50_: inhibition of XO 50% activity (concentration in µg/mL required for a 50% reduction in XO activity).

## Data Availability

Data are contained within the article and Supplementary Materials.

## Supplementary Materials

The following supporting information can be downloaded at: https://www.mdpi.com/article/10.3390/molecules29184468/s1, Figures S1–S48 shows 1D NMR of compounds **1**–**24**; Table S1: Molecular docking parameters.
