# Supplementary material for: Bioassay-Guided Isolation and Identification of Xanthine Oxidase Inhibitory Constituents from the Fruits of Chaenomeles speciosa (Sweet) Nakai
Source: Molecules. 2024 Sep 20;29(18):4468. doi: 10.3390/molecules29184468 (PMC11434067; doi:10.3390/molecules29184468)
Supplement: Supplementary file 1 [file molecules-29-04468-s001.zip › molecules-3210676-supplementary.pdf]

Supplementary Materials

Bioassay-Guided Isolation and Identification of Xanthine Oxidase Inhibitory Constituents from the fruits of *Chaenomeles speciosa* (Sweet) Nakai

Kui Li [1], Ruoling Xu [1], Mengting Kuang, Wei Ma and Ning Li *

Anhui Key Laboratory of Bioactivity of Natural Products, School of Pharmacy, Anhui Medical University, Hefei 230032, China; lkui0912@foxmail.com (K.L.); xuruoling0126@gmail.com (R.X.); kuangmengting@ahmu.edu.cn (M.K.); mw421553449@sina.com (W.M.);

1 These authors contributed equally to this work.

* Correspondence: 1993500019@ahmu.edu.cn (N.L.); Tel.: +86-551-6516115 (N.L.)

Table of Contents

1D-NMR spectra of compounds **1** S3

1D-NMR spectra of compounds **2** S4

1D-NMR spectra of compounds **3** S5

1D-NMR spectra of compounds **4** S6

1D-NMR spectra of compounds **5** S7

1D-NMR spectra of compounds **6** S8

1D-NMR spectra of compounds **7** S9

1D-NMR spectra of compounds **8** S10

1D-NMR spectra of compounds **9** S11

1D-NMR spectra of compounds **10** S12

1D-NMR spectra of compounds **11** S13

1D-NMR spectra of compounds **12** S14

1D-NMR spectra of compounds **13** S15

1D-NMR spectra of compounds **14** S16

1D-NMR spectra of compounds **15** S17

1D-NMR spectra of compounds **16** S18

1D-NMR spectra of compounds **17** S19

1D-NMR spectra of compounds **18** S20

1D-NMR spectra of compounds **19** S21

1D-NMR spectra of compounds **20** S22

1D-NMR spectra of compounds **21** S23

1D-NMR spectra of compounds **22** S24

1D-NMR spectra of compounds **23** S25

1D-NMR spectra of compounds **24** S26

1D-NMR data of compounds **1-24** S27-32

Table S1: molecular docking parameters S33-34

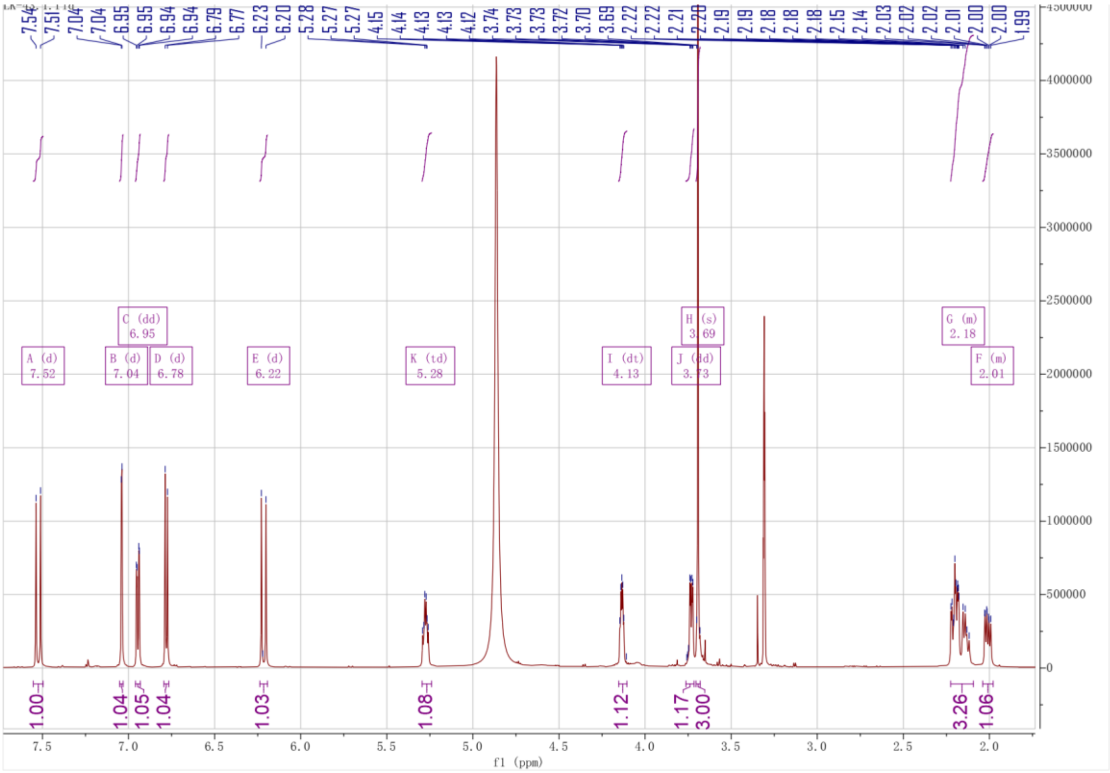

Fig. S1. ^{1}H-NMR spectrum of compound **1** (600 MHz, CD_3OD)

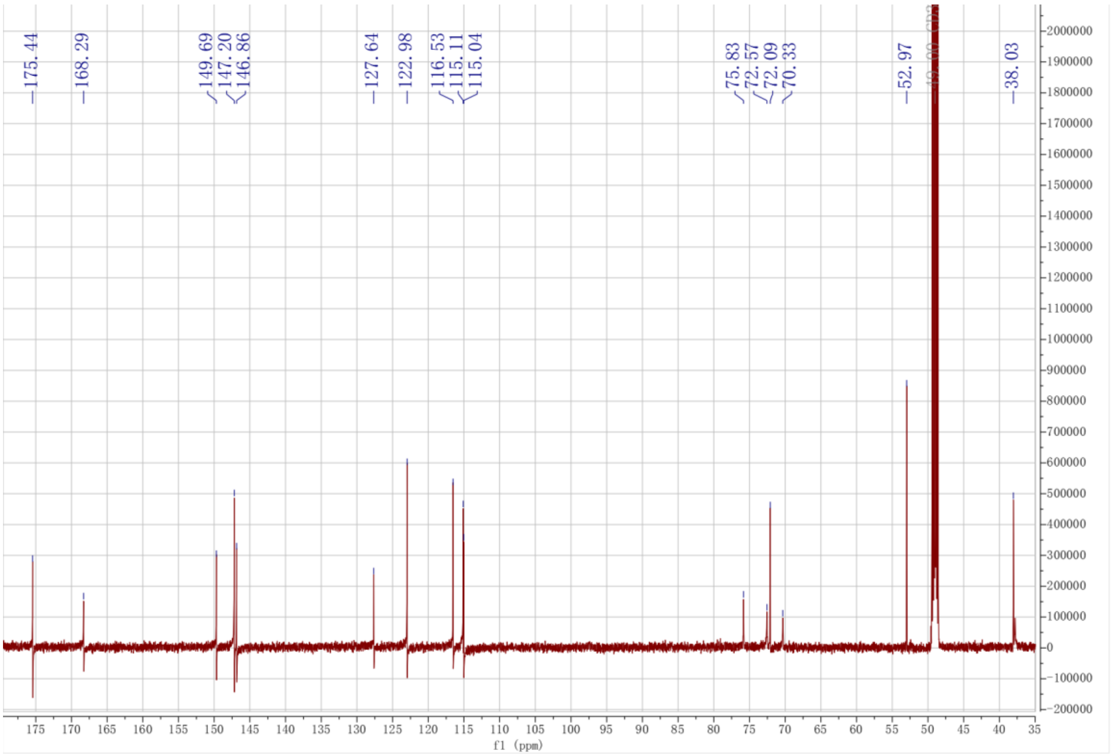

Fig. S2. ^{13}C-NMR spectra of compound **1** (151 MHz, CD_3OD)

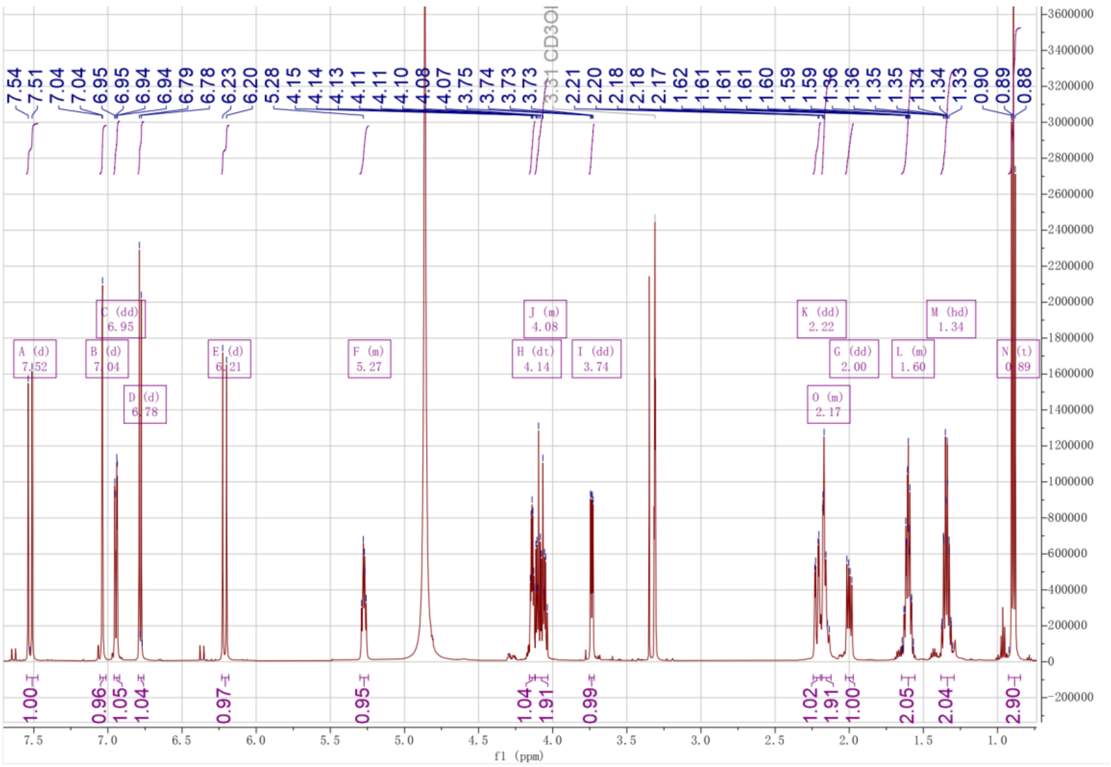

Fig. S3. ^{1}H-NMR spectrum of compound **2** (600 MHz, CD_3OD)

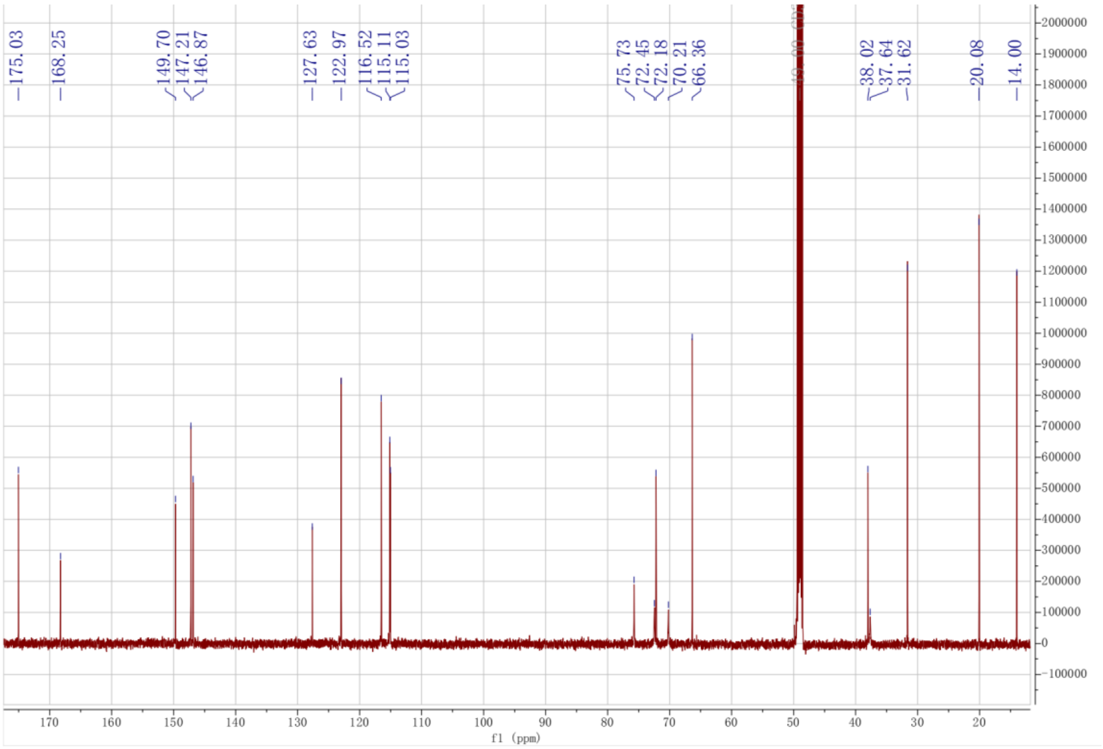

Fig. S4. ^{13}C-NMR spectra of compound **2** (151 MHz, CD_3OD)

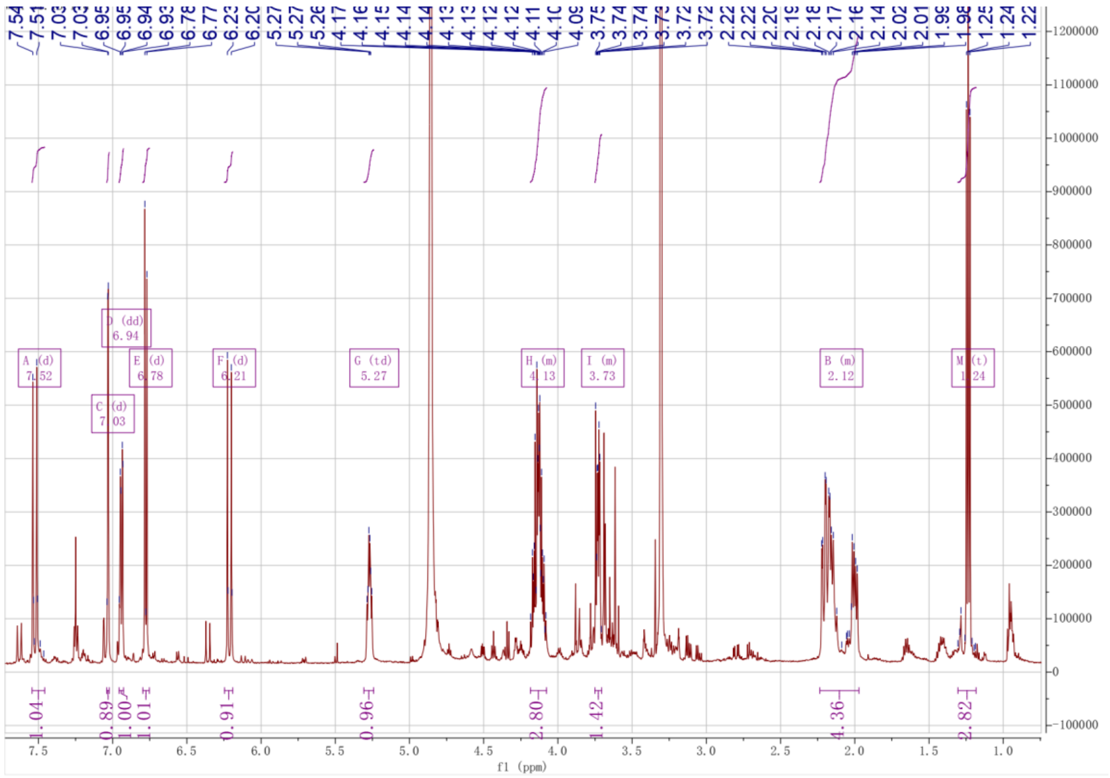

Fig. S5. ^{1}H-NMR spectrum of compound **3** (600 MHz, CD_3OD)

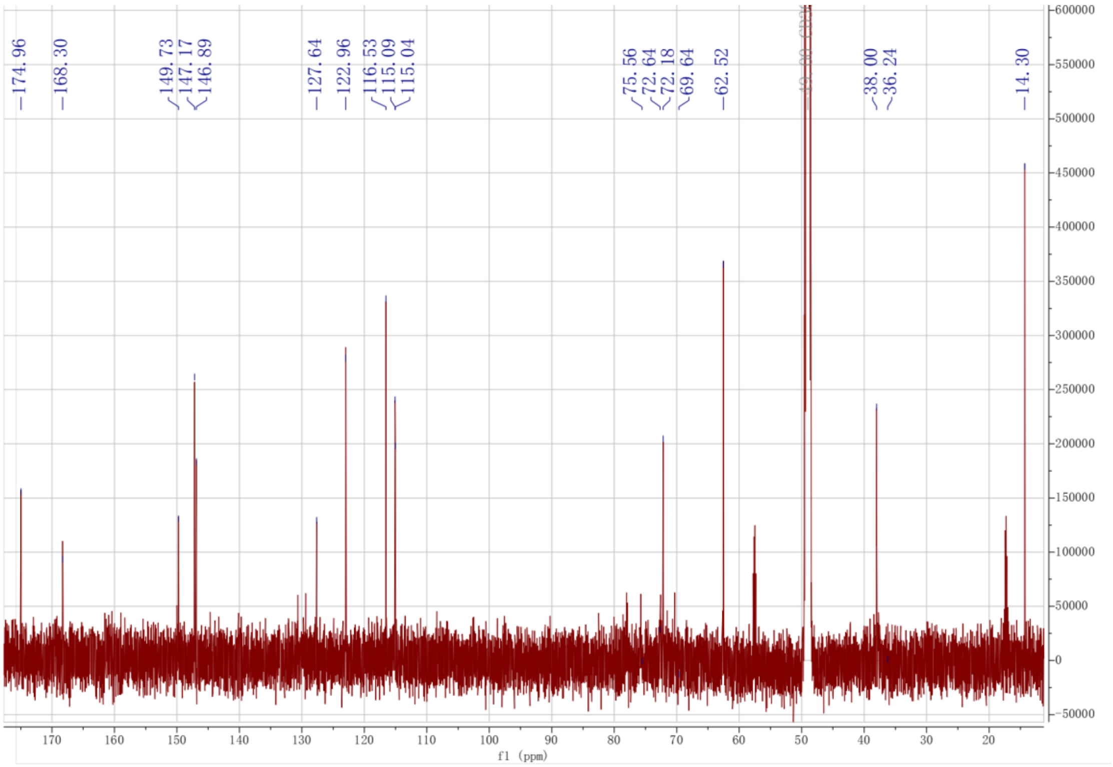

Fig. S6. ^{13}C-NMR spectra of compound **3** (151 MHz, CD_3OD)

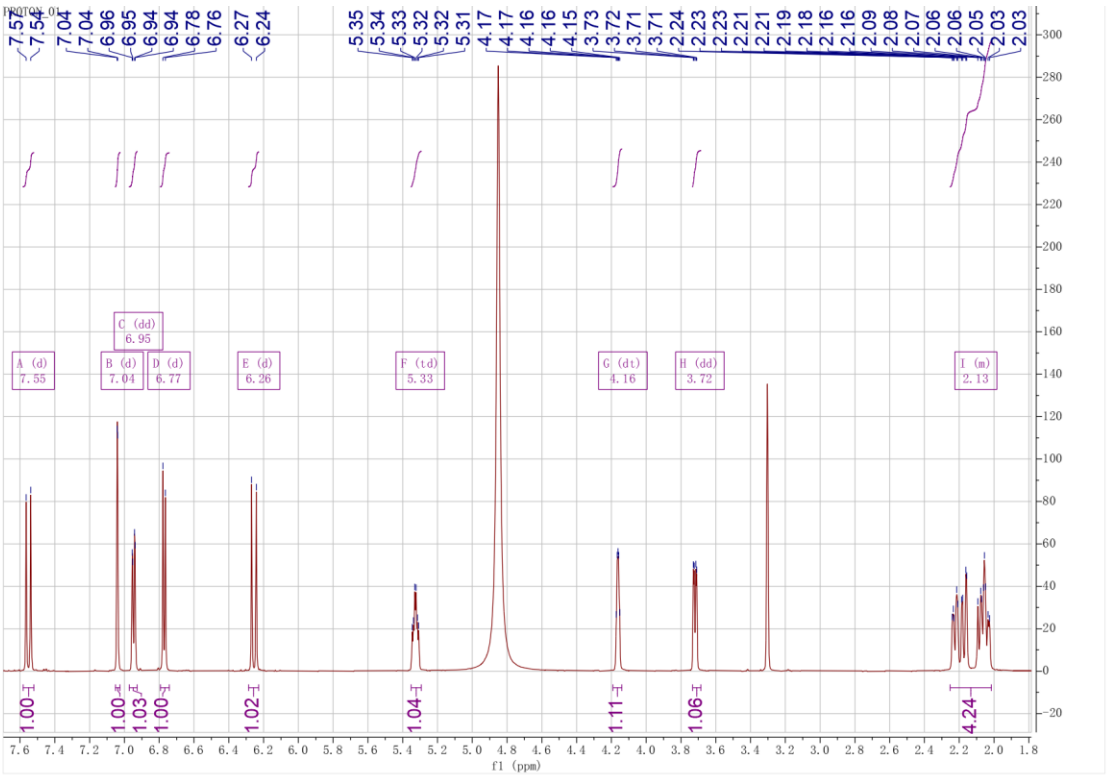

Fig. S7. ^{1}H-NMR spectrum of compound **4** (600 MHz, CD_3OD)

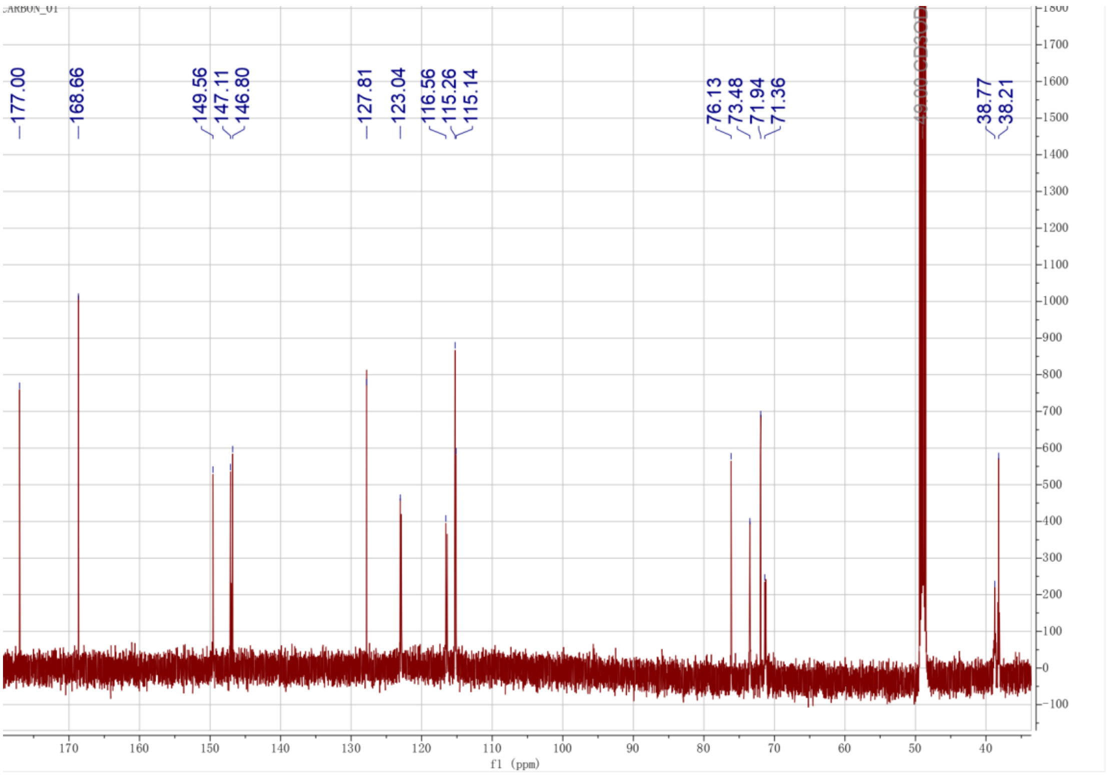

Fig. S8. ^{13}C-NMR spectra of compound **4** (151 MHz, CD_3OD)

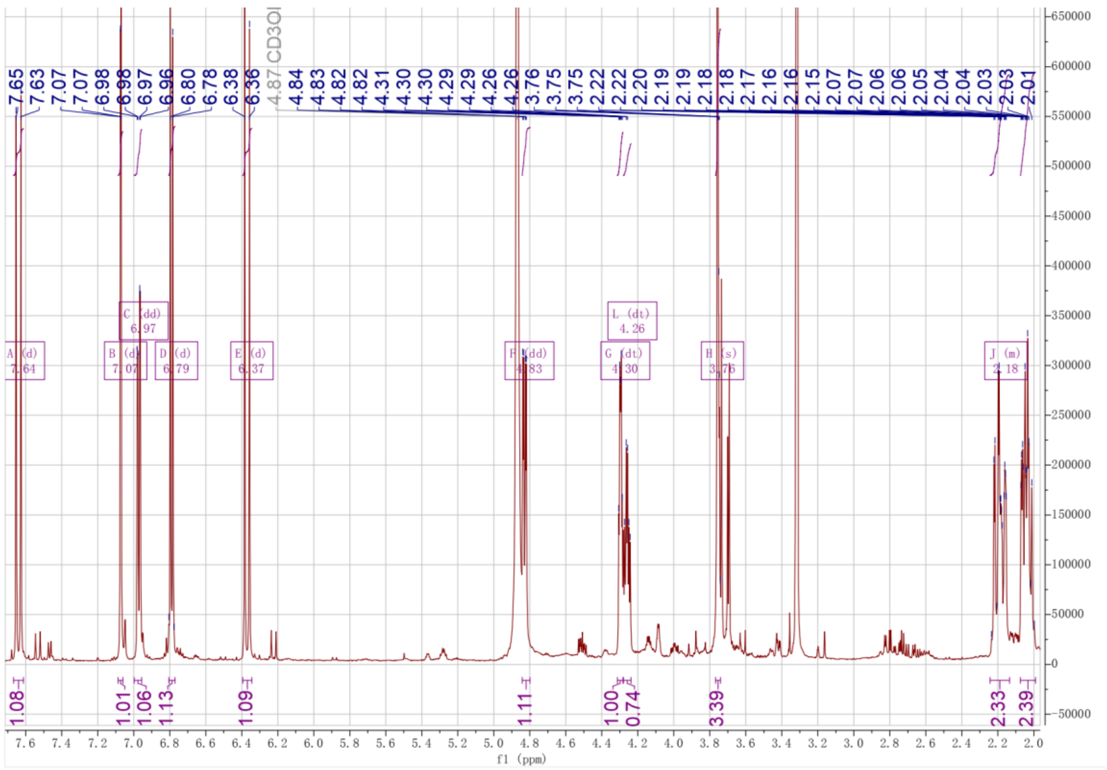

Fig. S9. ^{1}H-NMR spectrum of compound **5** (600 MHz, CD_3OD)

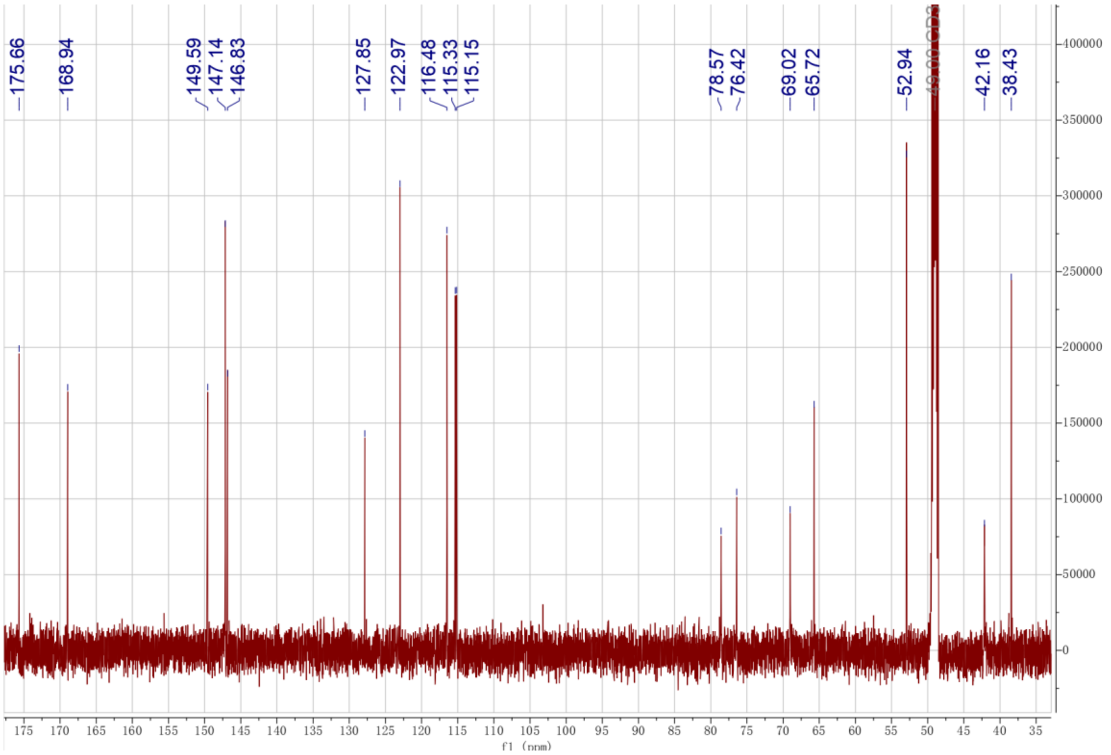

Fig. S10. ^{13}C-NMR spectra of compound **5**(151 MHz, CD_3OD)

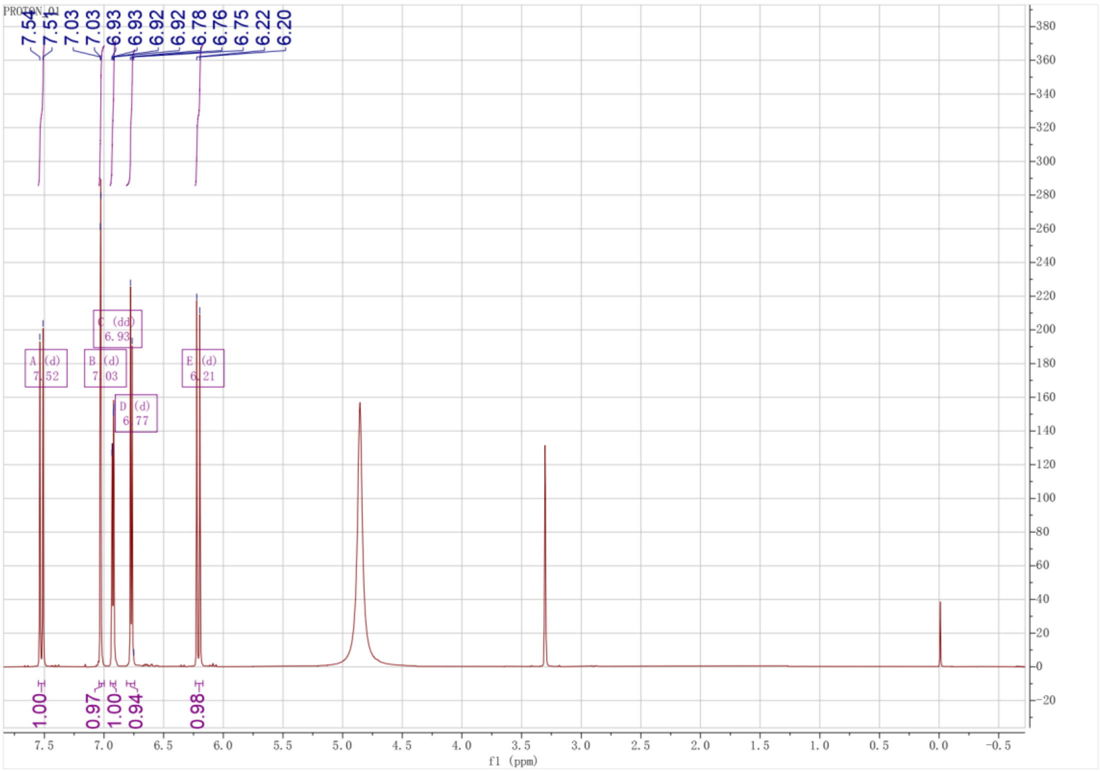

Fig. S11. ^{1}H-NMR spectrum of compound **5** (600 MHz, CD_3OD)

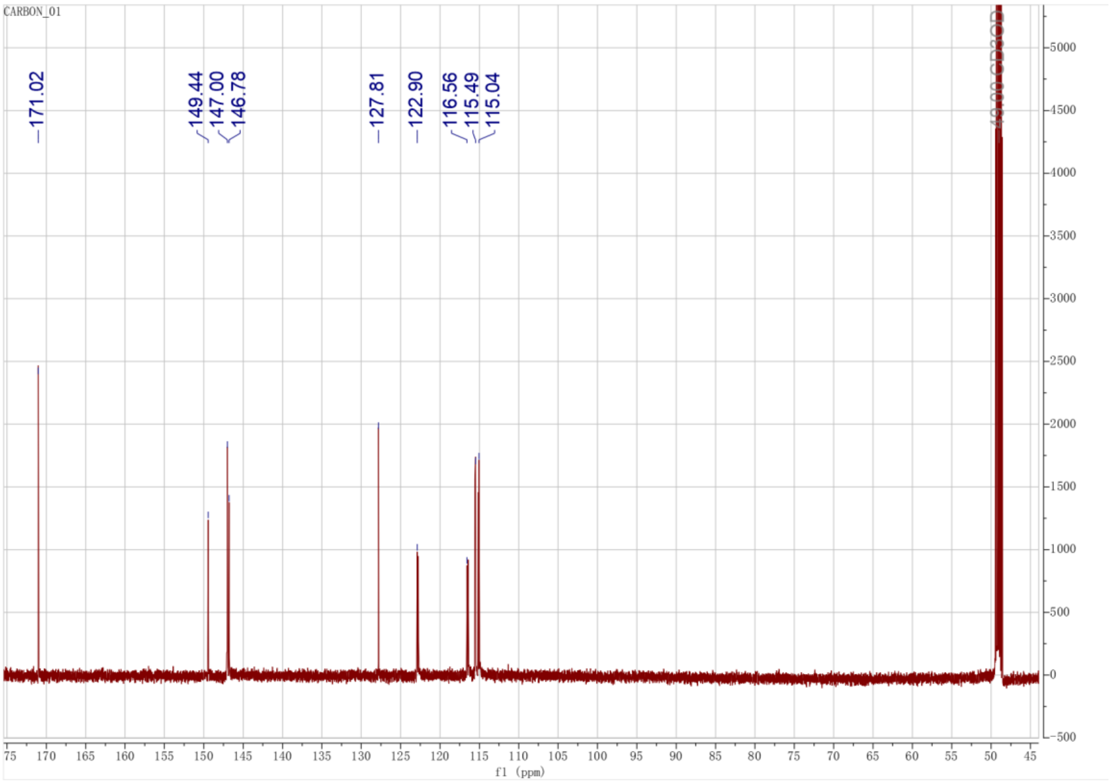

Fig. S12. ^{13}C-NMR spectra of compound **6**(151 MHz, CD_3OD)

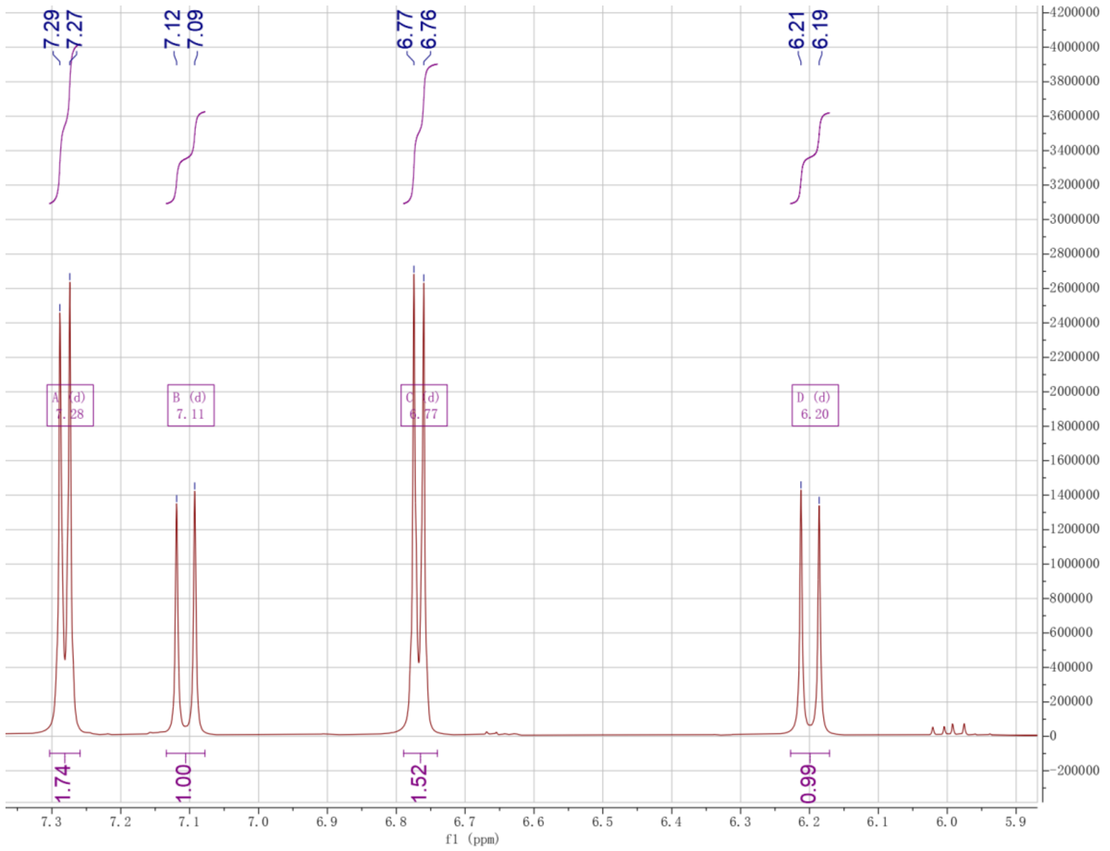

Fig. S13. ^{1}H-NMR spectrum of compound **6** (600 MHz, DMSO-d_6)

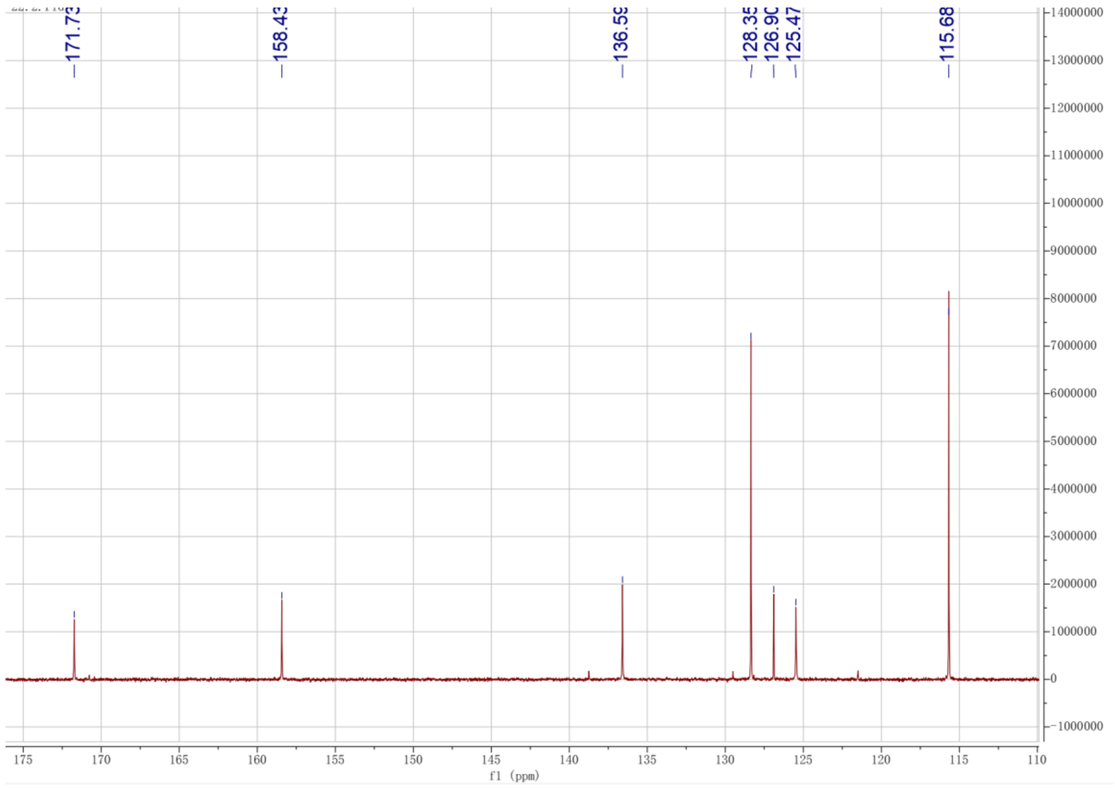

Fig. S14. ^{13}C-NMR spectra of compound **6** (151 MHz, DMSO-d_6)

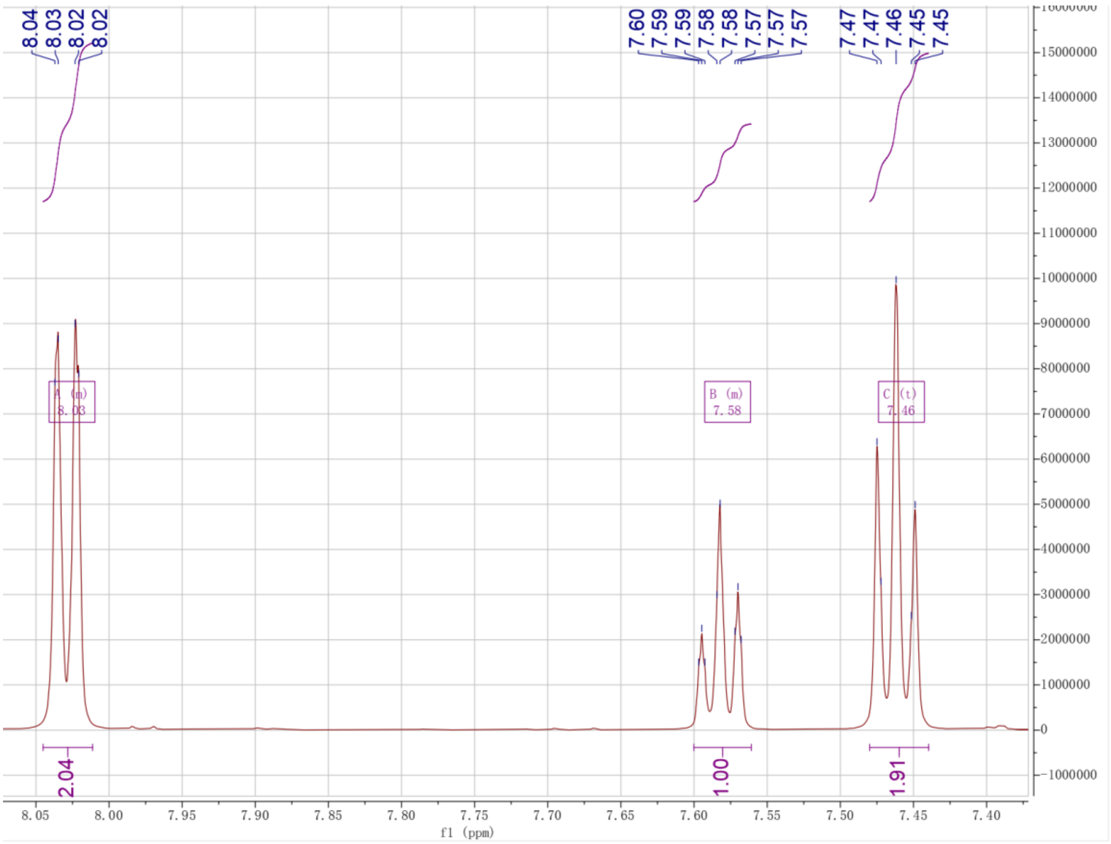

Fig. S15. ^{1}H-NMR spectrum of compound **8** (600 MHz, CD_3OD)

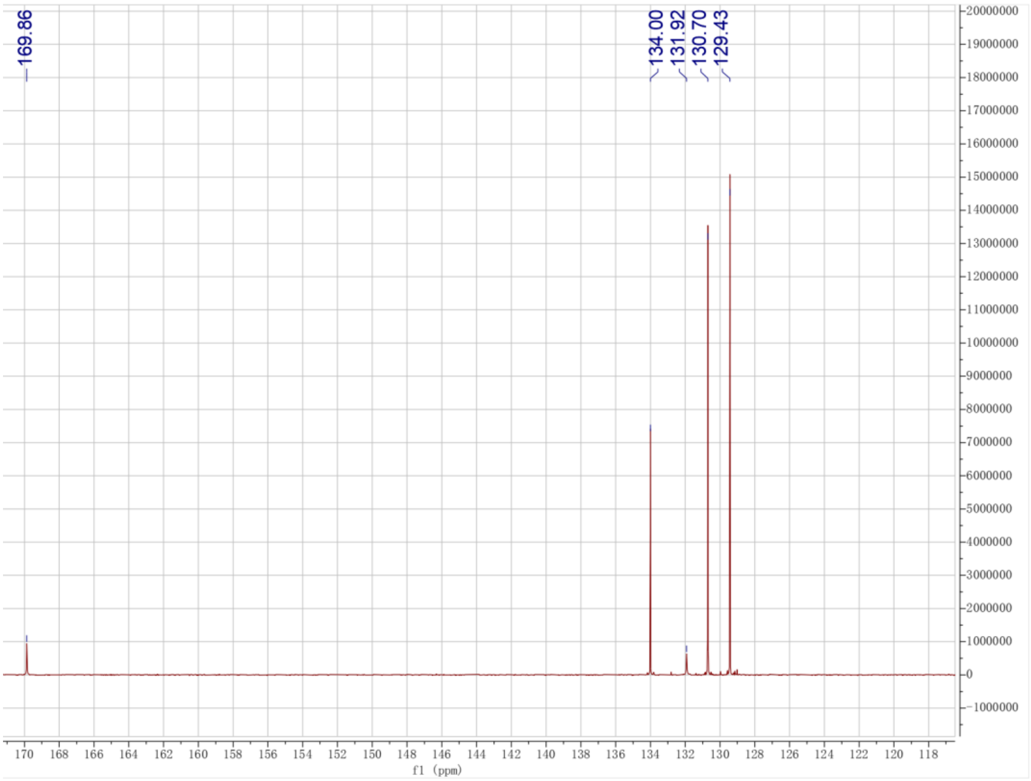

Fig. S16. ^{13}C-NMR spectra of compound **8** (151 MHz, CD_3OD)

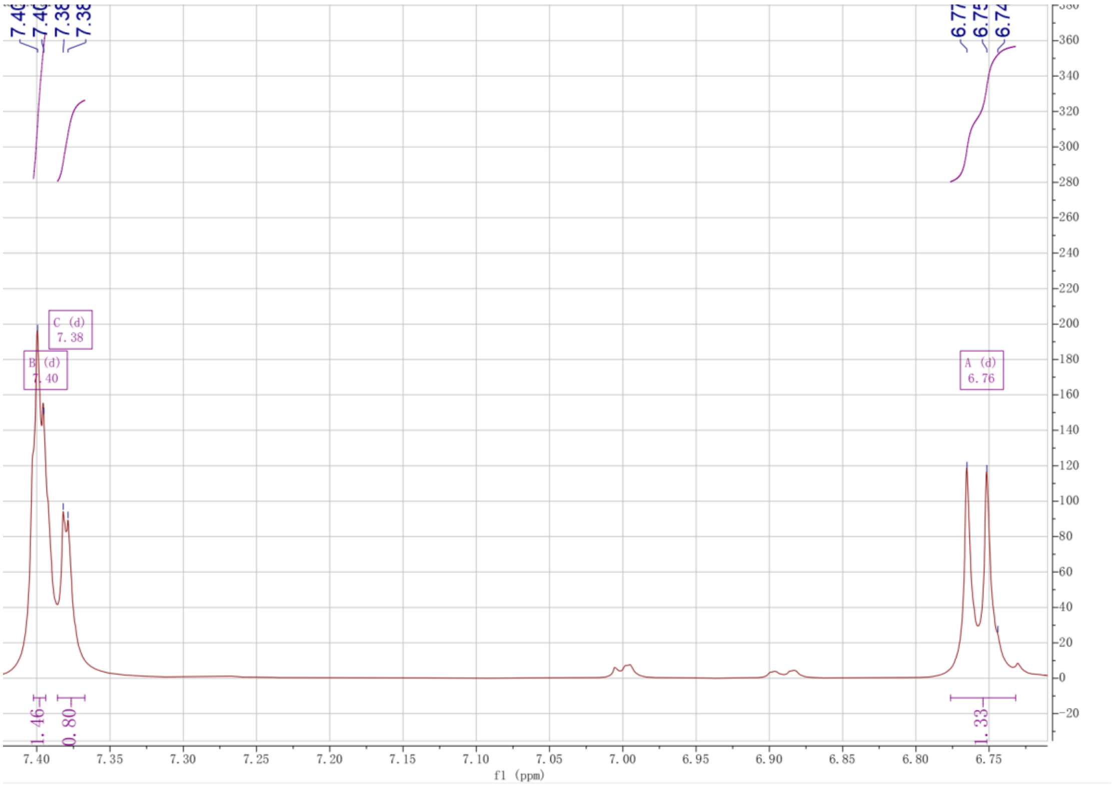

Fig. S17. ^{1}H-NMR spectrum of compound **9** (600 MHz, CD_3OD)

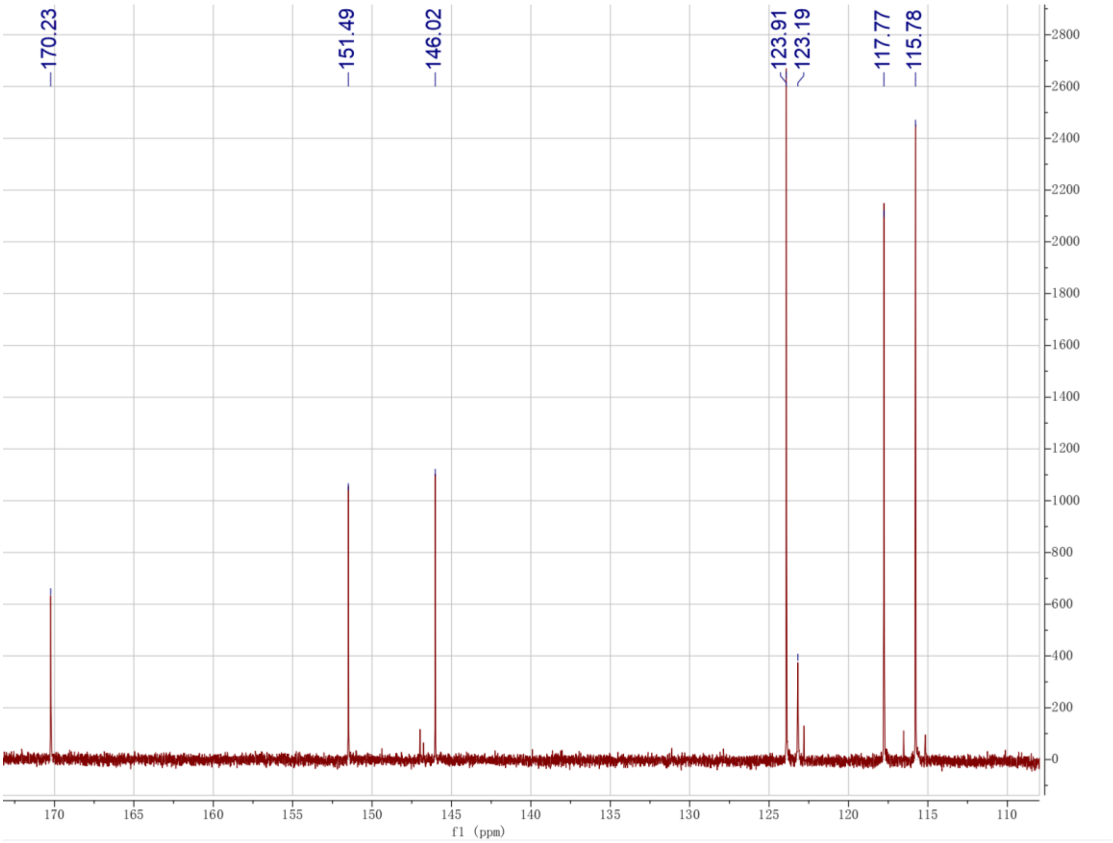

Fig. S18. ^{13}C-NMR spectra of compound **9** (151 MHz, CD_3OD)

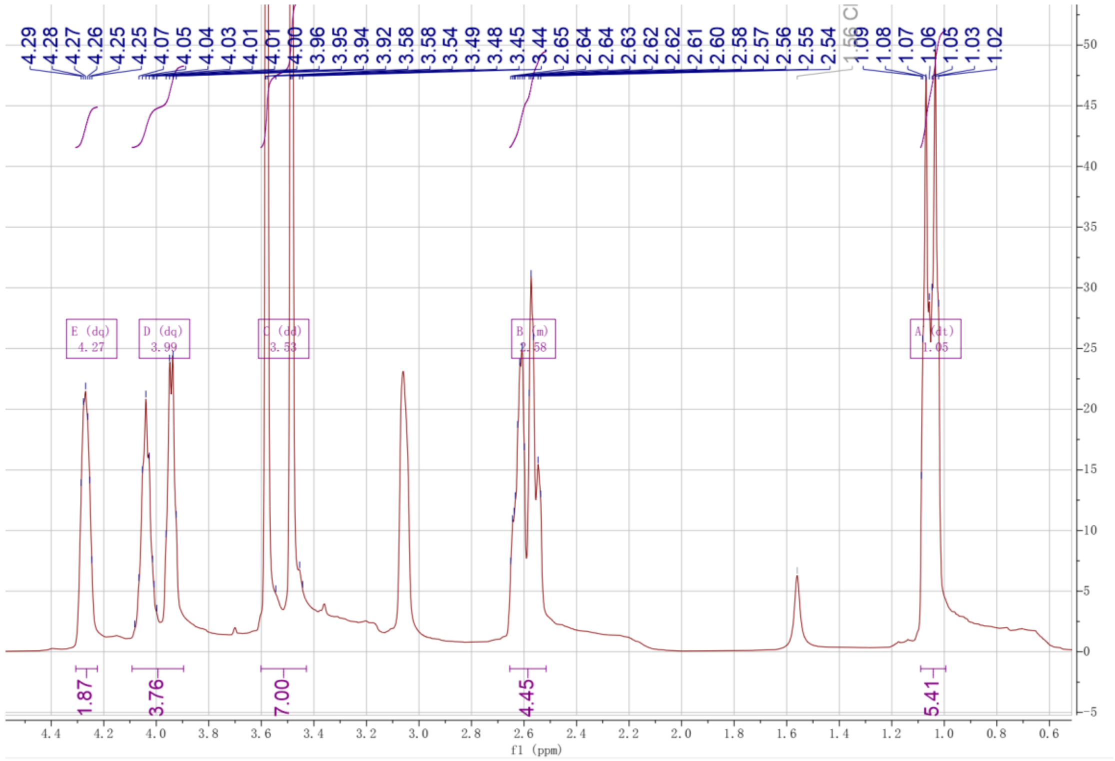

Fig. S19. ^{1}H-NMR spectrum of compound **10a/10b** (600 MHz, $CDCl_3$)

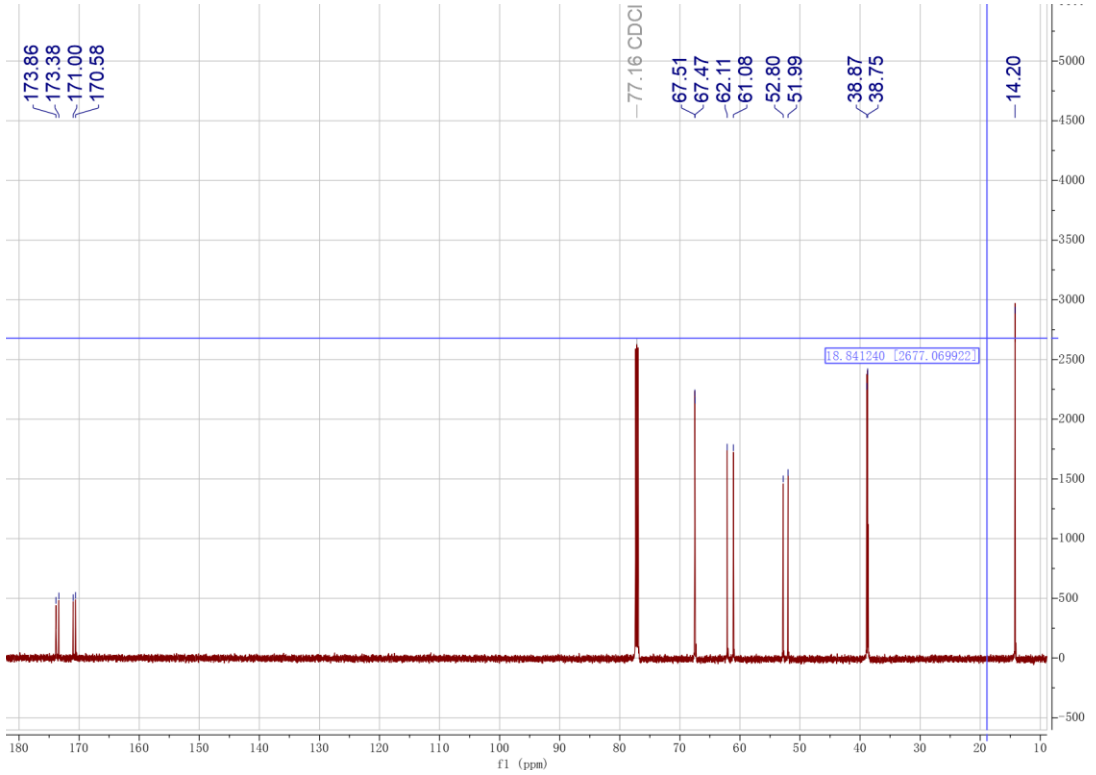

Fig. S20. ^{13}C-NMR spectra of compound **10a/10b** (151 MHz, $CDCl_3$)

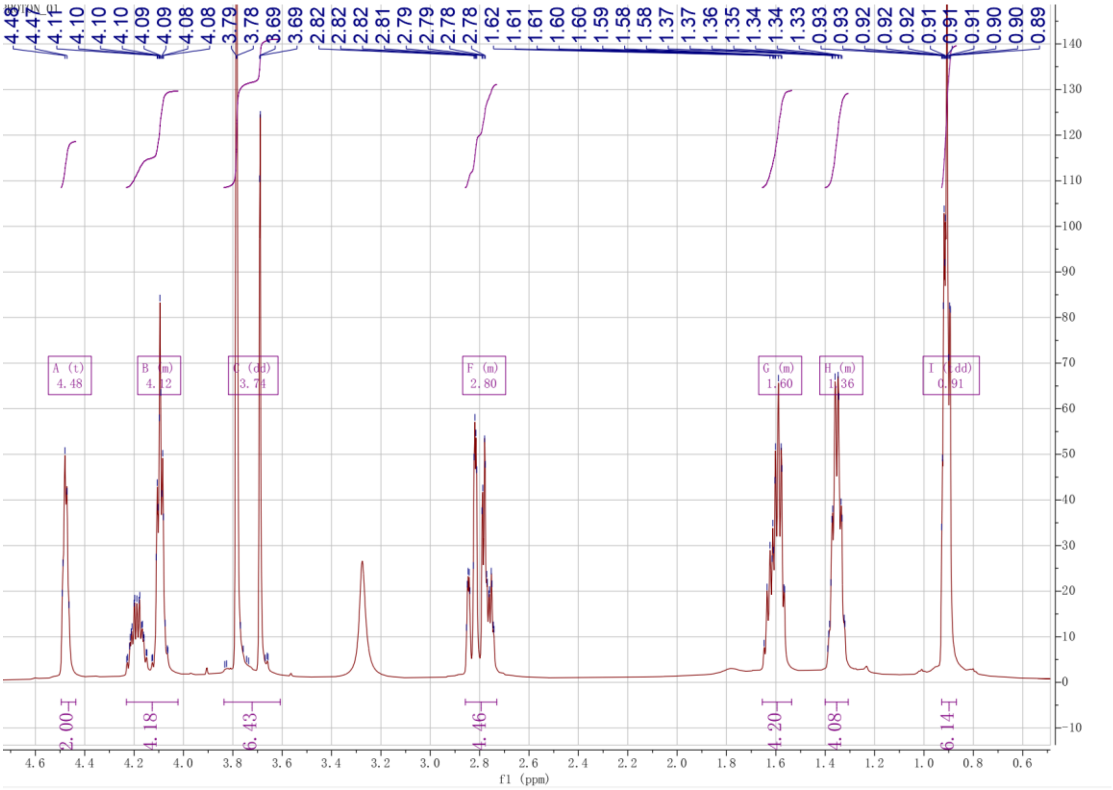

Fig. S21. ^{1}H-NMR spectrum of compound **11a/11b** (600 MHz, $CDCl_3$)

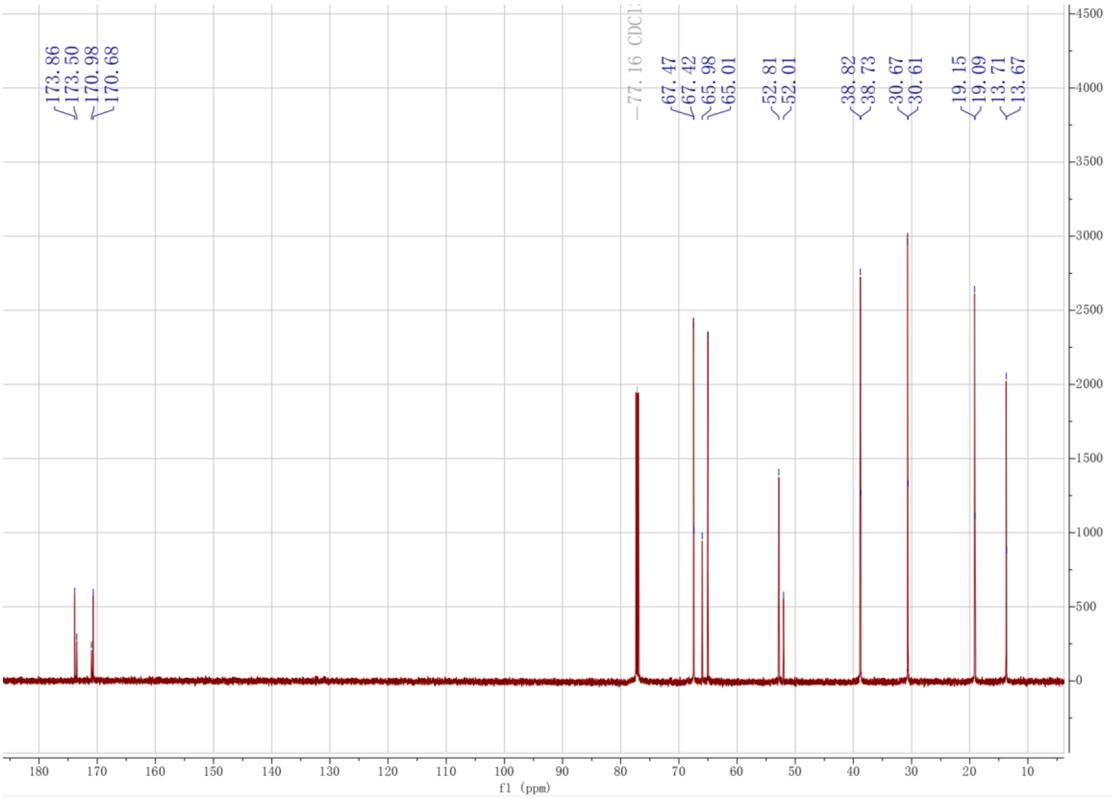

Fig. S22. ^{13}C-NMR spectra of compound **11a/11b** (151 MHz, $CDCl_3$)

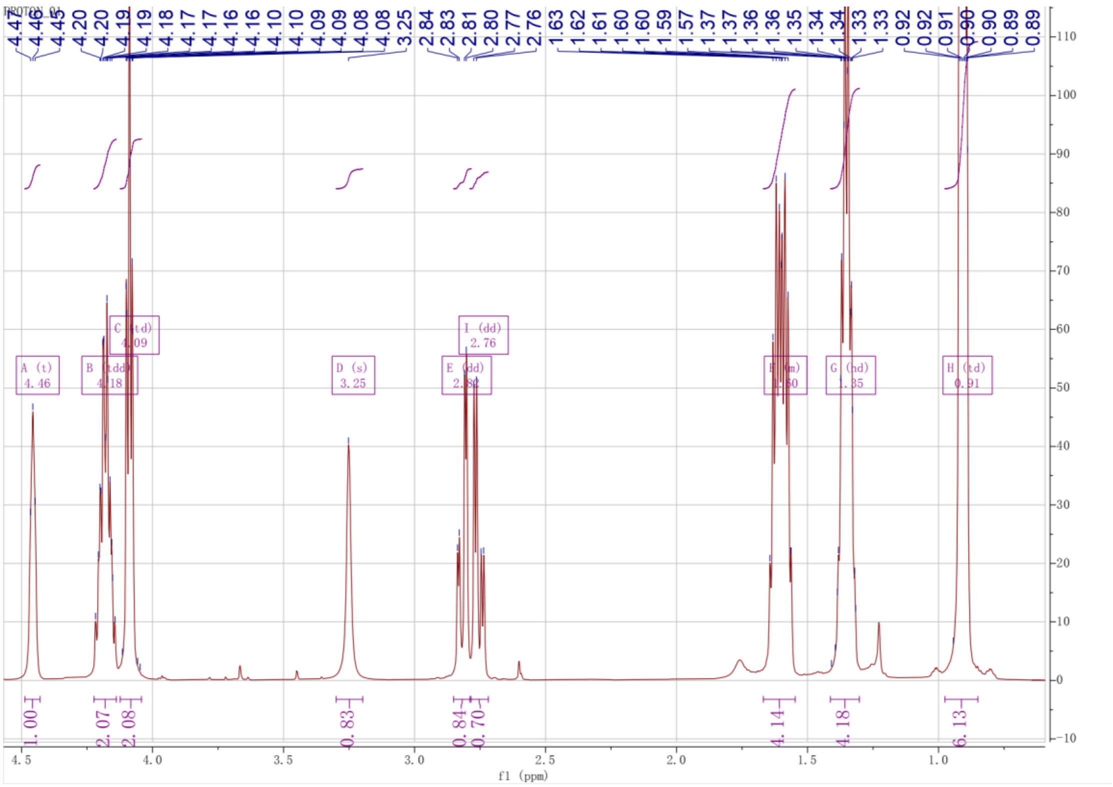

Fig. S23. ^{1}H-NMR spectrum of compound **12** (600 MHz, $CDCl_3$)

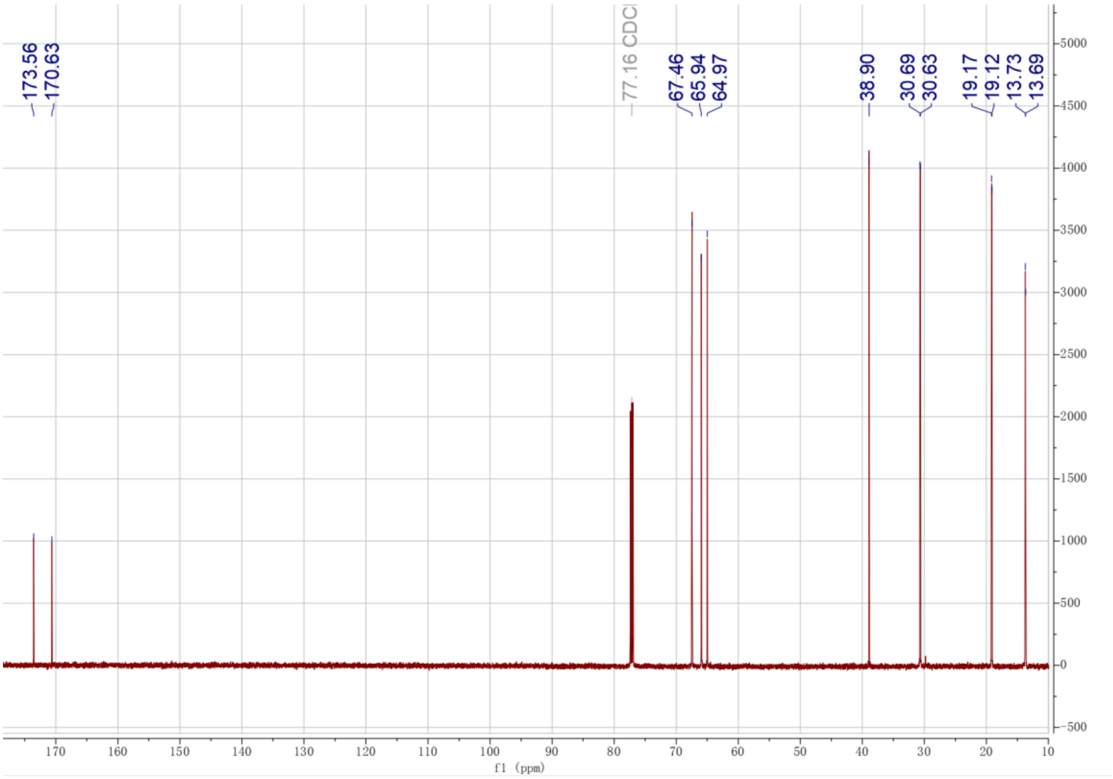

Fig. S24. ^{13}C-NMR spectra of compound **12** (151 MHz, $CDCl_3$)

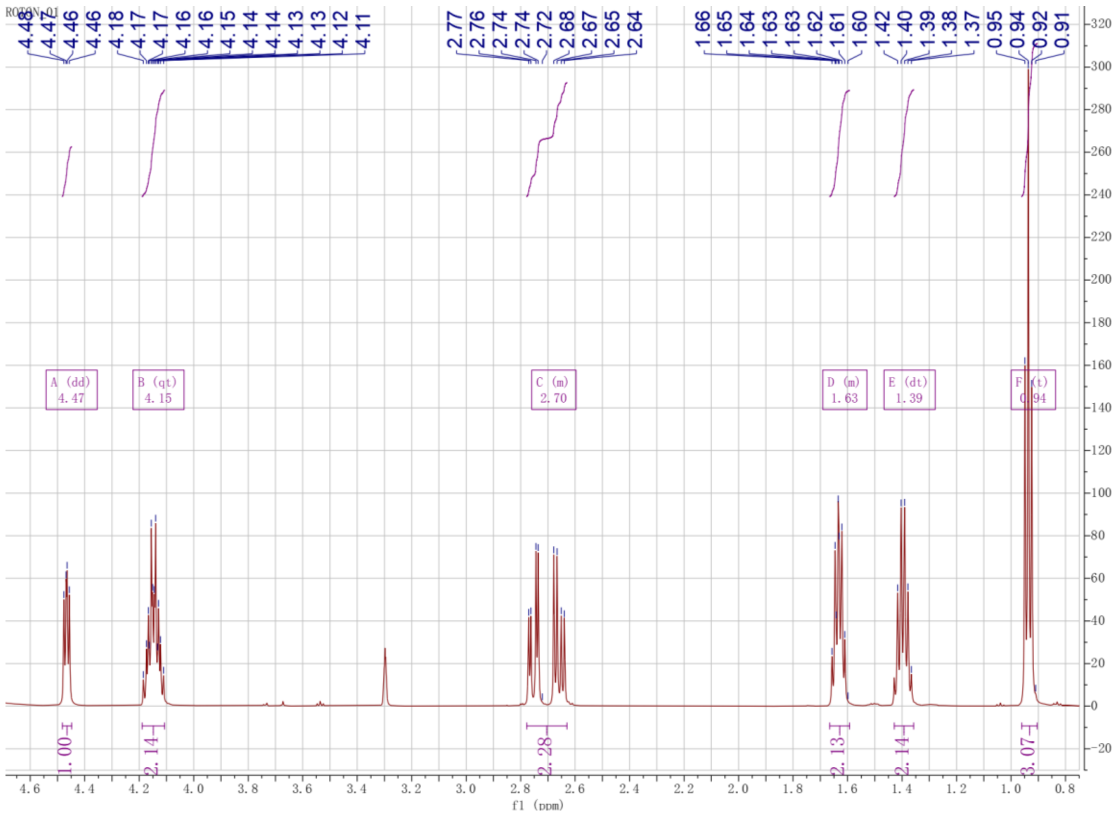

Fig. S25. ^{1}H-NMR spectrum of compound **13** (600 MHz, CD_3OD)

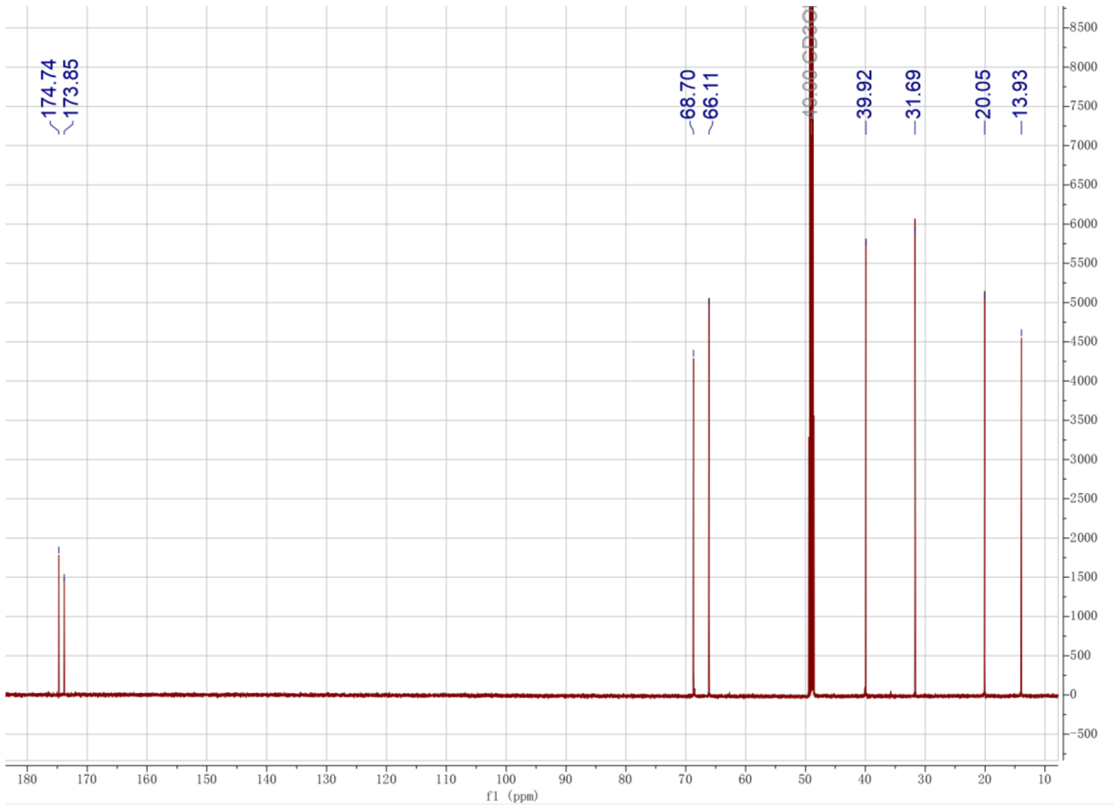

Fig. S26. ^{13}C-NMR spectra of compound **13** (151 MHz, CD_3OD)

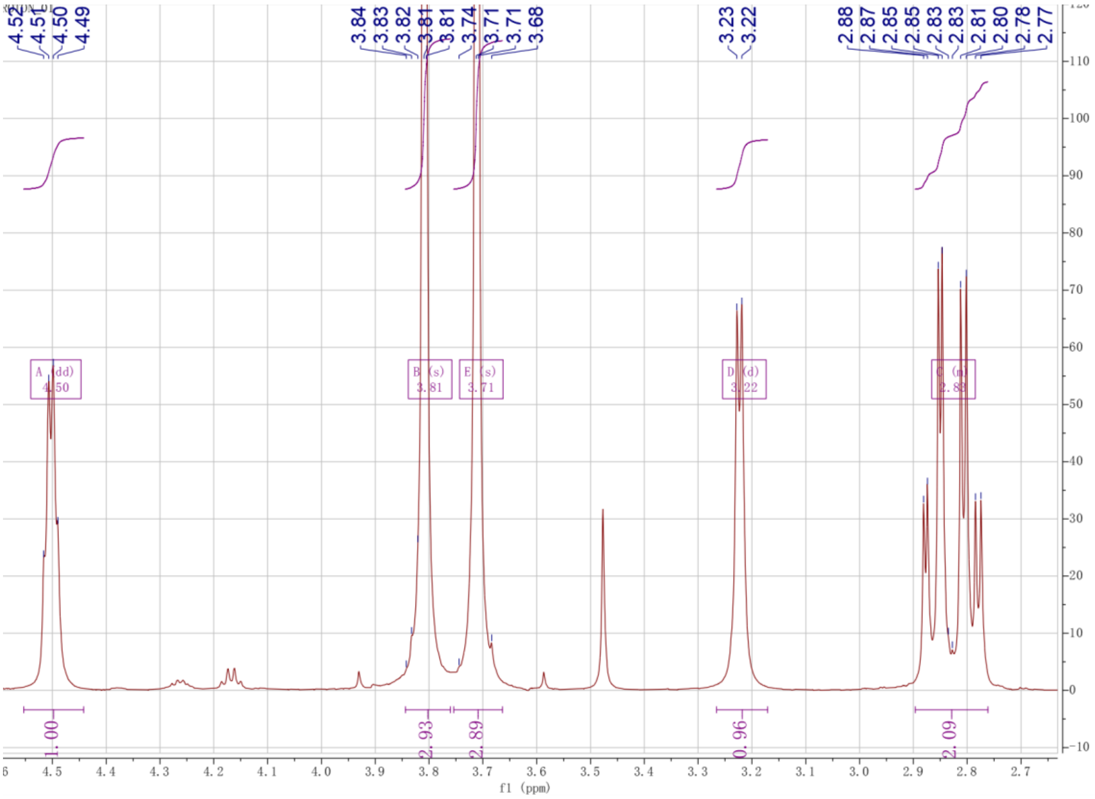

Fig. S27. ^{1}H-NMR spectrum of compound **14** (600 MHz, $CDCl_3$)

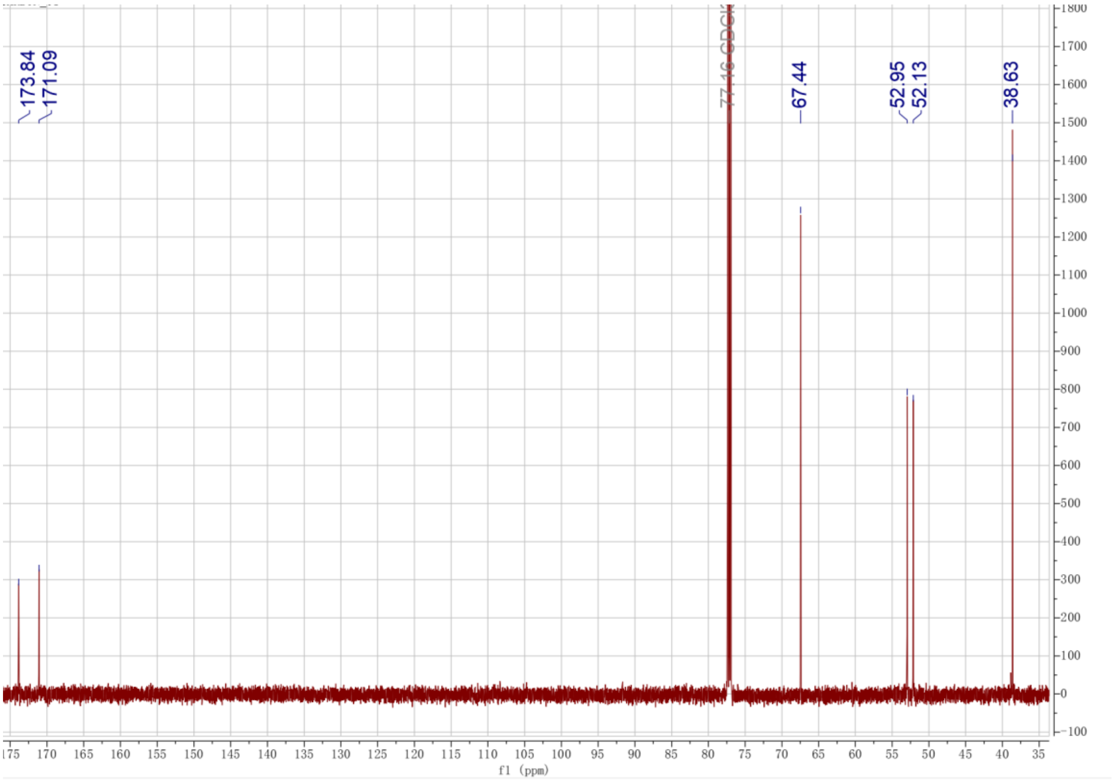

Fig. S28. ^{13}C-NMR spectra of compound **14** (151 MHz, $CDCl_3$)

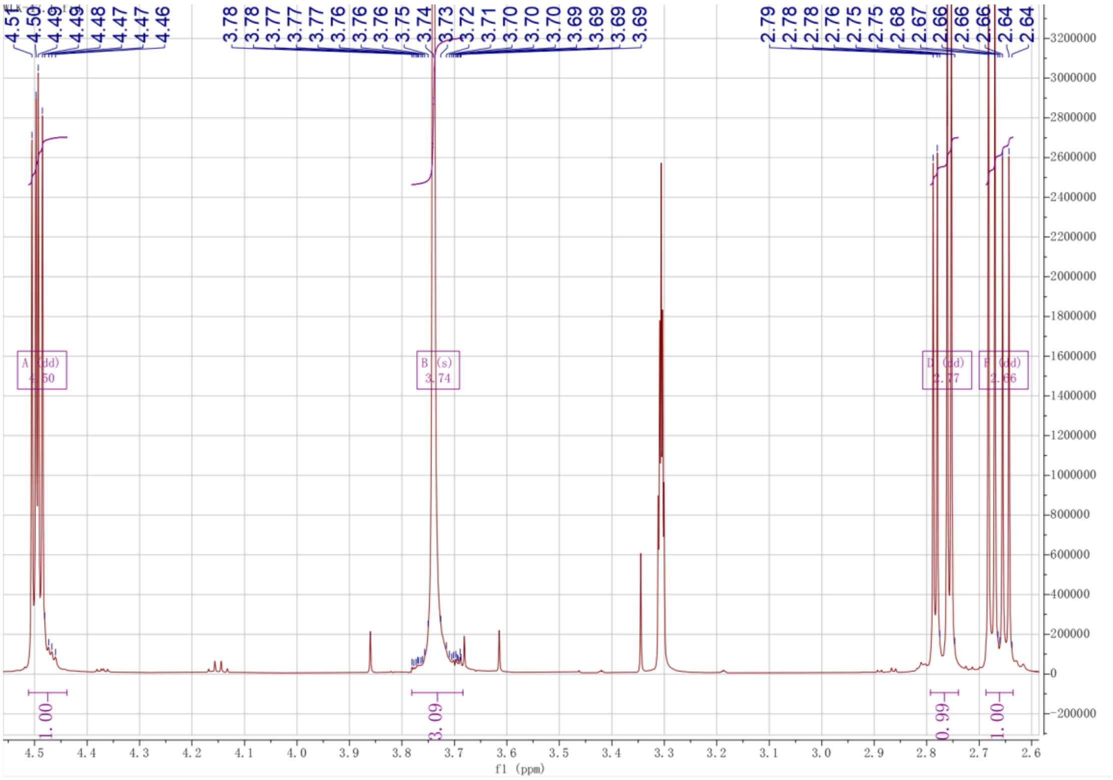

Fig. S29. ^{1}H-NMR spectrum of compound **15** (600 MHz, CD_3OD)

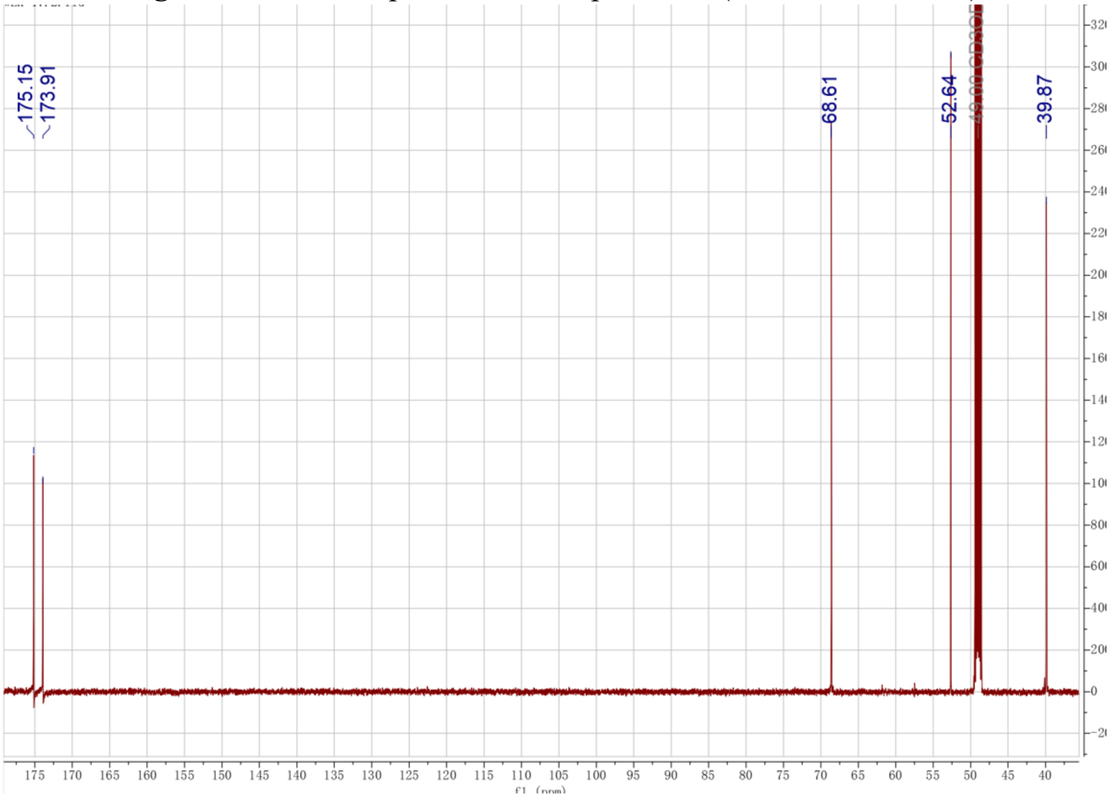

Fig. S30. ^{13}C-NMR spectra of compound **15** (151 MHz, CD_3OD)

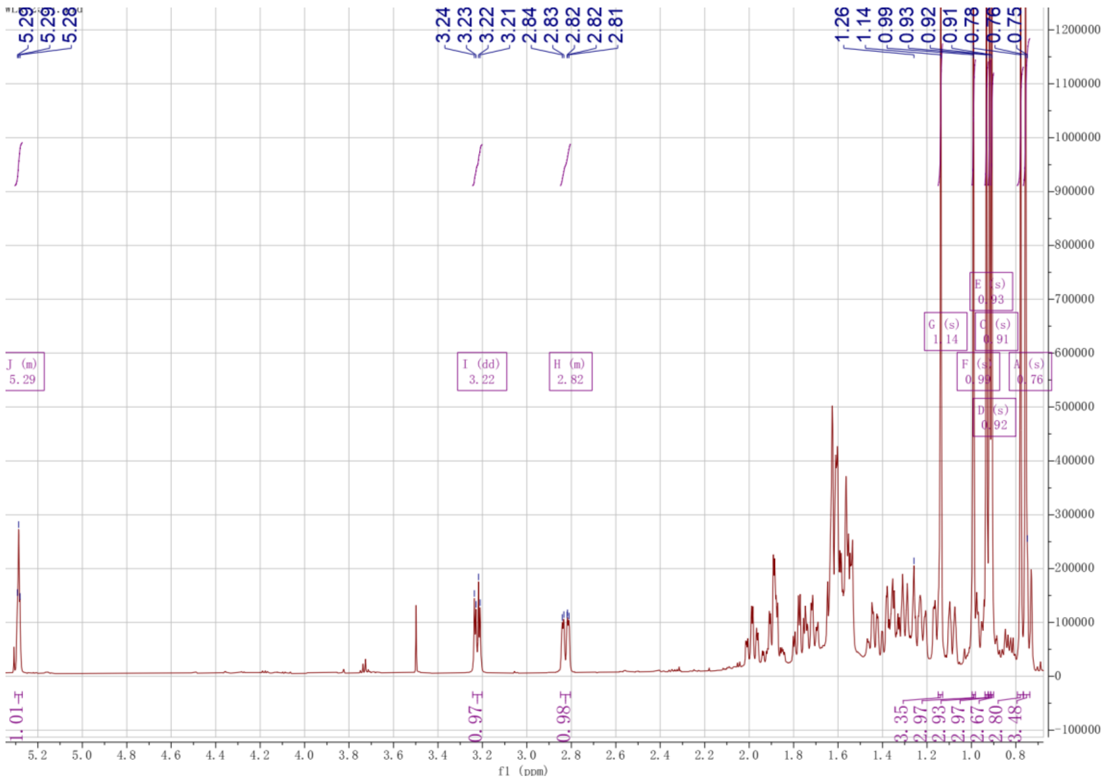

Fig. S31. ^{1}H-NMR spectrum of compound **16** (600 MHz, $CDCl_3$)

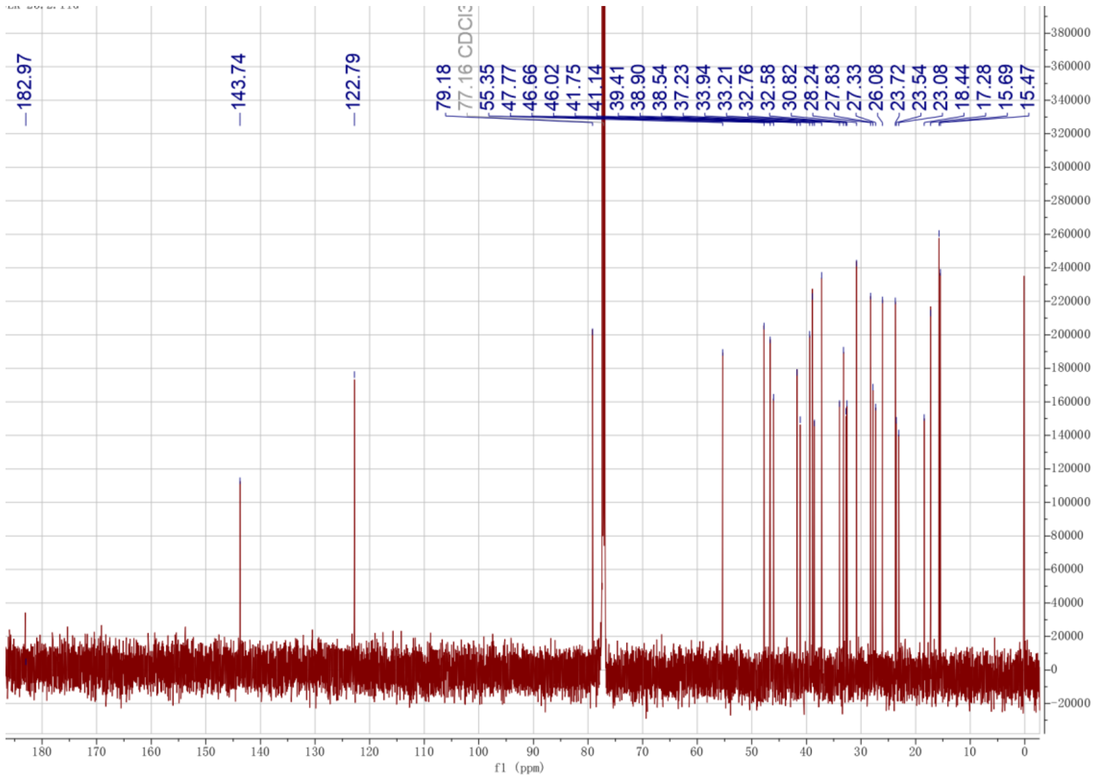

Fig. S32. ^{13}C-NMR spectra of compound **16** (151 MHz, $CDCl_3$)

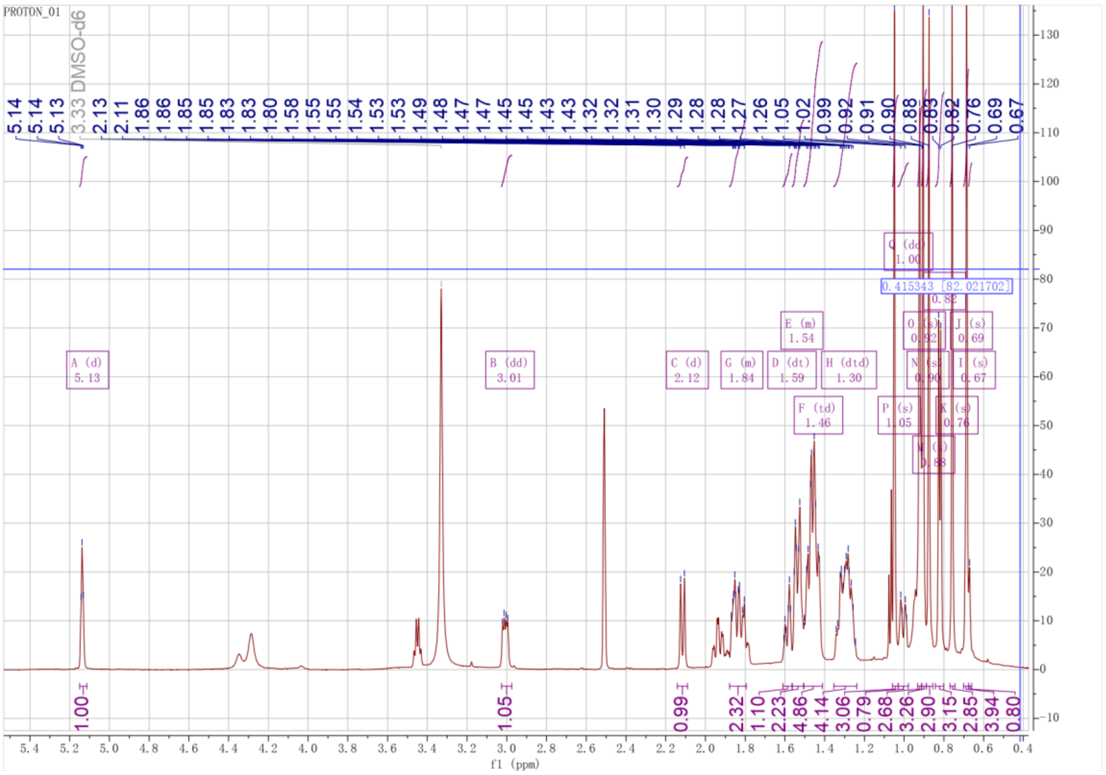

Fig. S33. ^{1}H-NMR spectrum of compound **17** (600 MHz, DMSO-d_6)

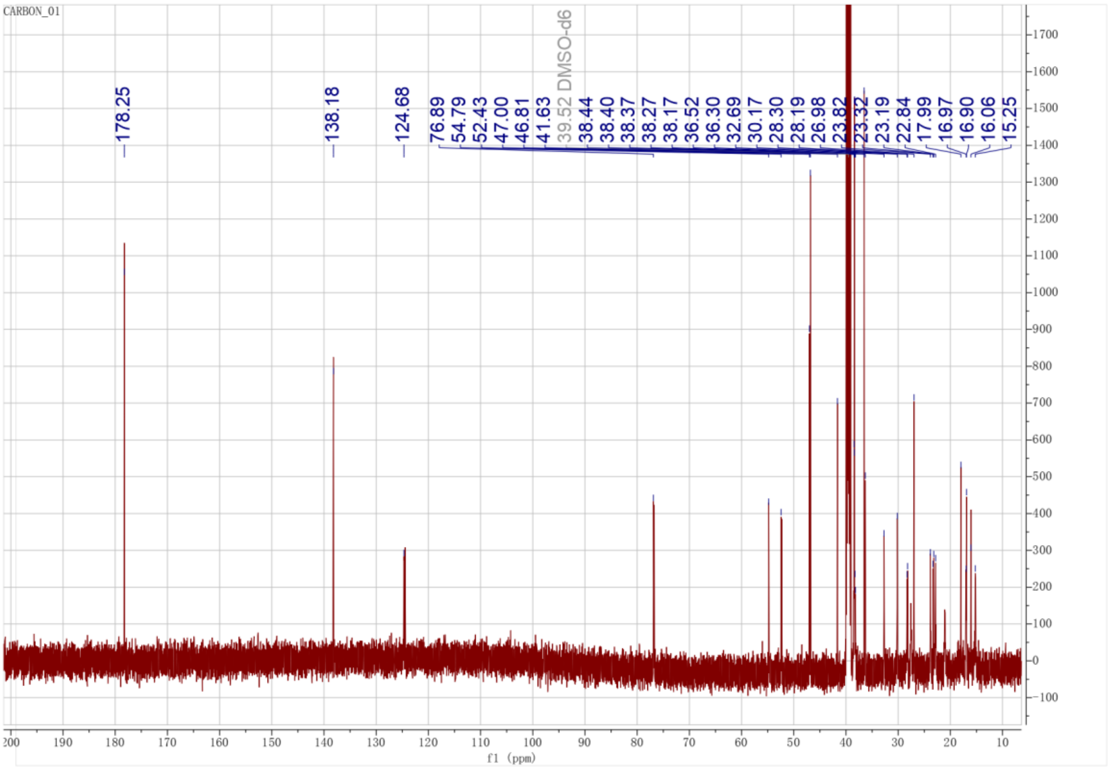

Fig. S34. ^{13}C-NMR spectra of compound **17** (151 MHz, DMSO-d_6)

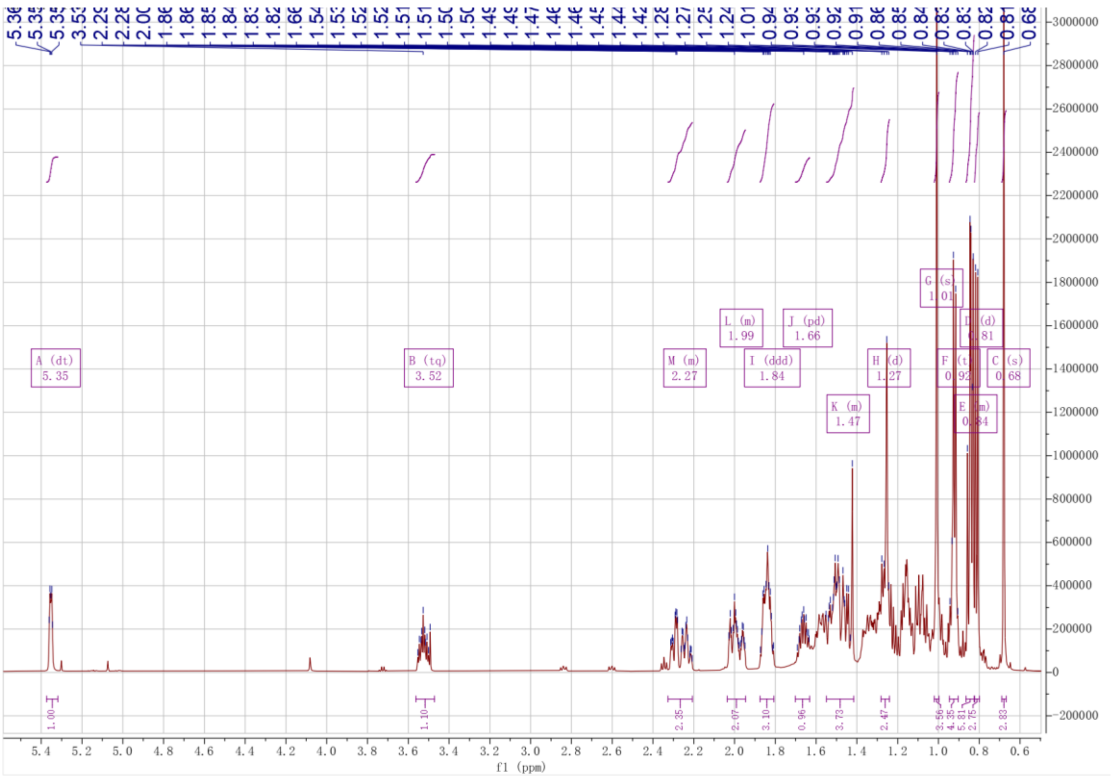

Fig. S35. ^{1}H-NMR spectrum of compound **18** (600 MHz, $CDCl_3$)

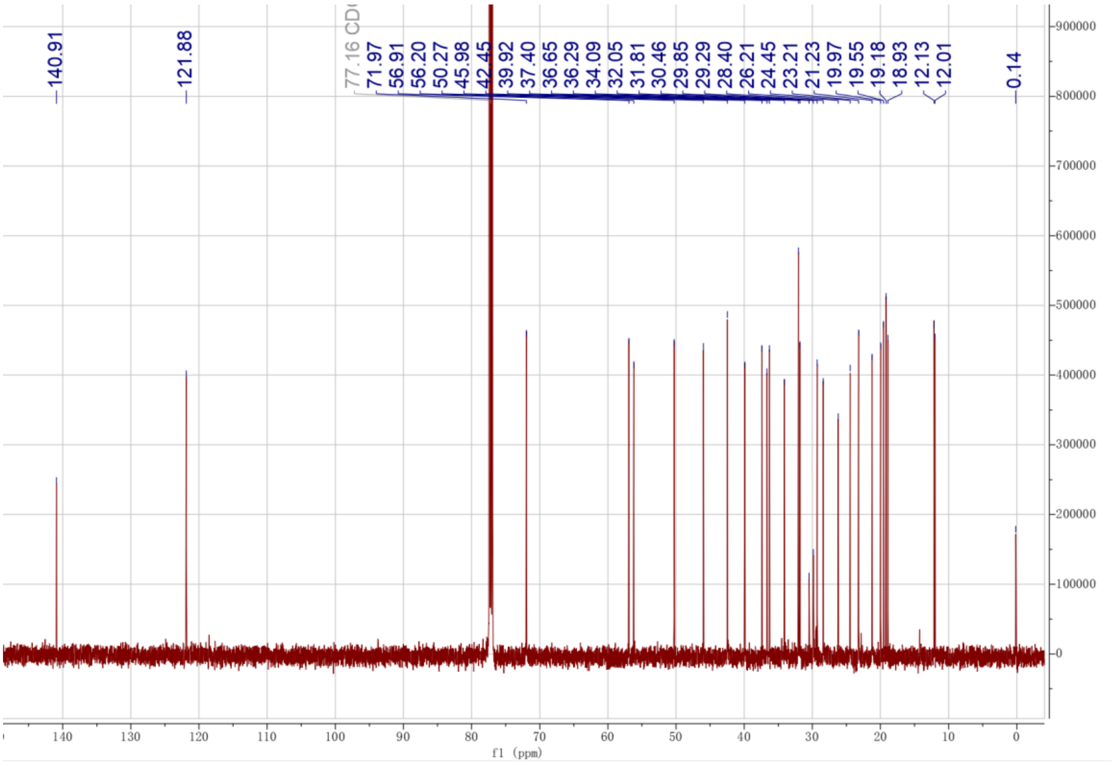

Fig. S36. ^{13}C-NMR spectra of compound **18** (151 MHz, $CDCl_3$)

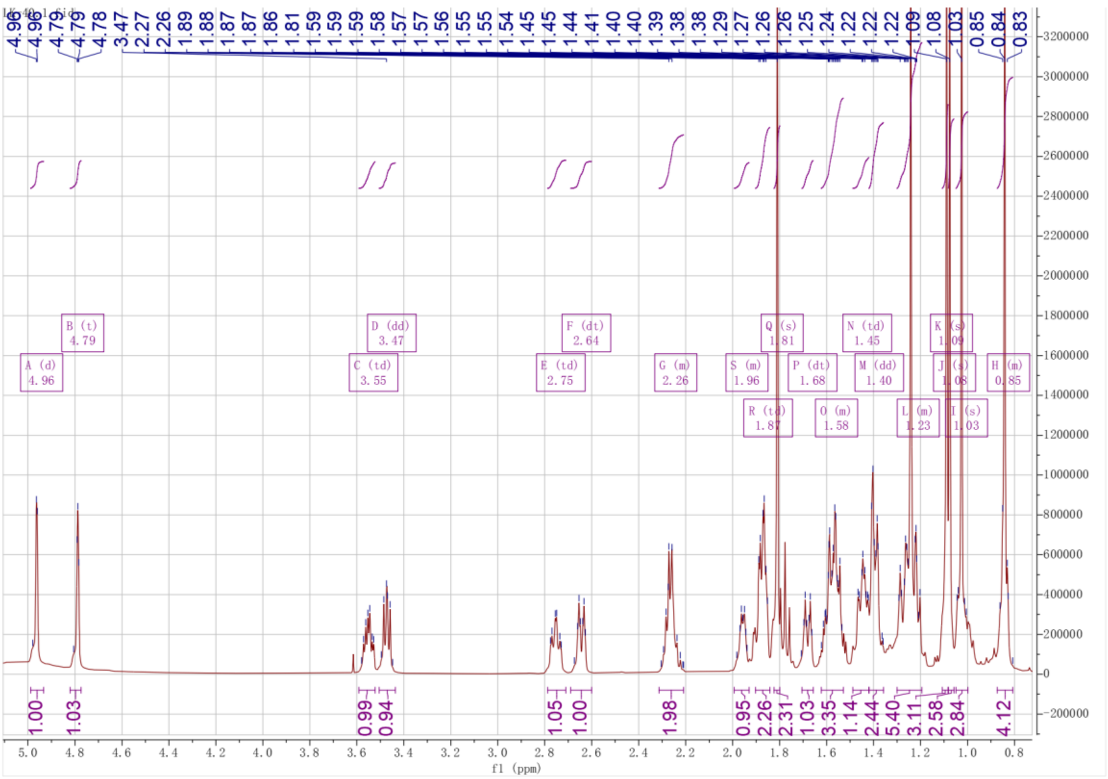

Fig. S37. ^{1}H-NMR spectrum of compound **19** (600 MHz, Pyridine-d_5)

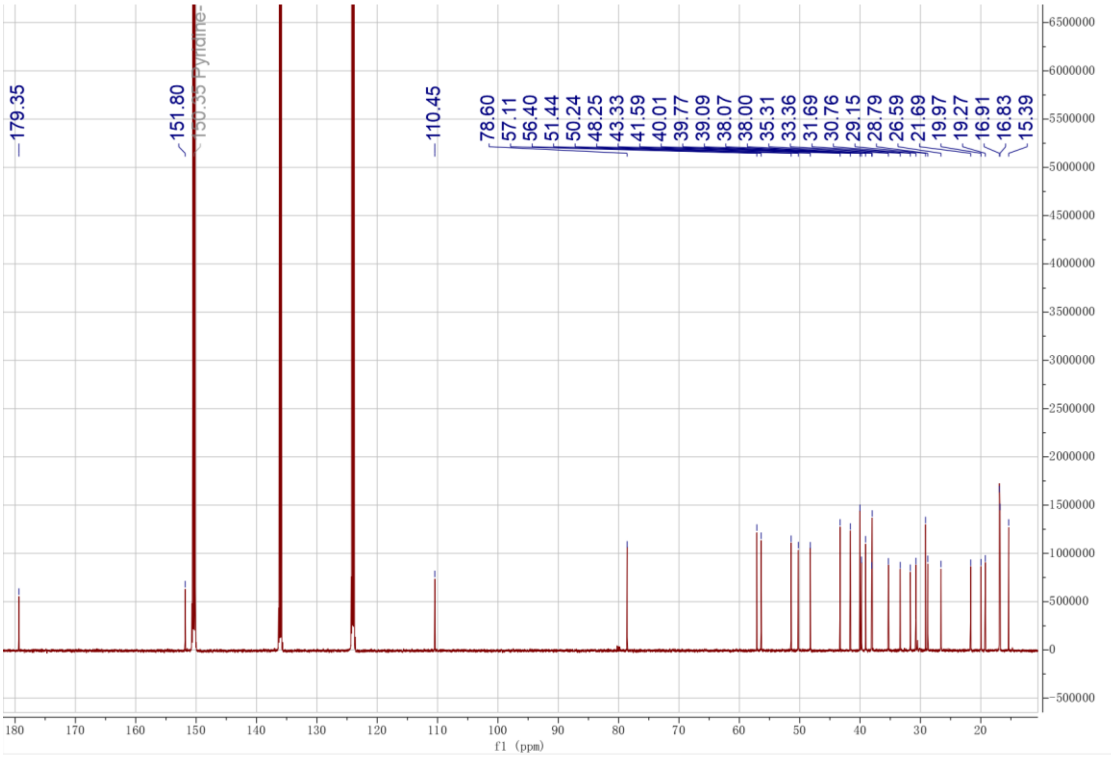

Fig. S38. ^{13}C-NMR spectra of compound **19** (151 MHz, Pyridine-d_5)

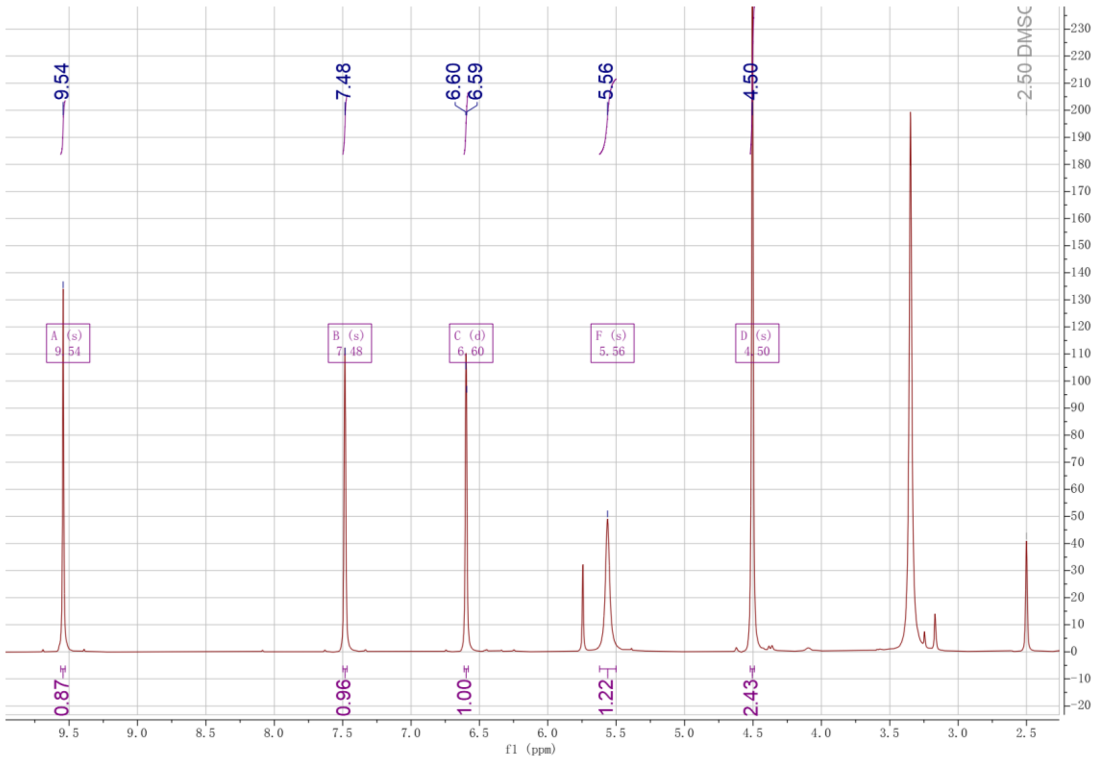

Fig. S39. ^{1}H-NMR spectrum of compound **20** (600 MHz, DMSO-d_6)

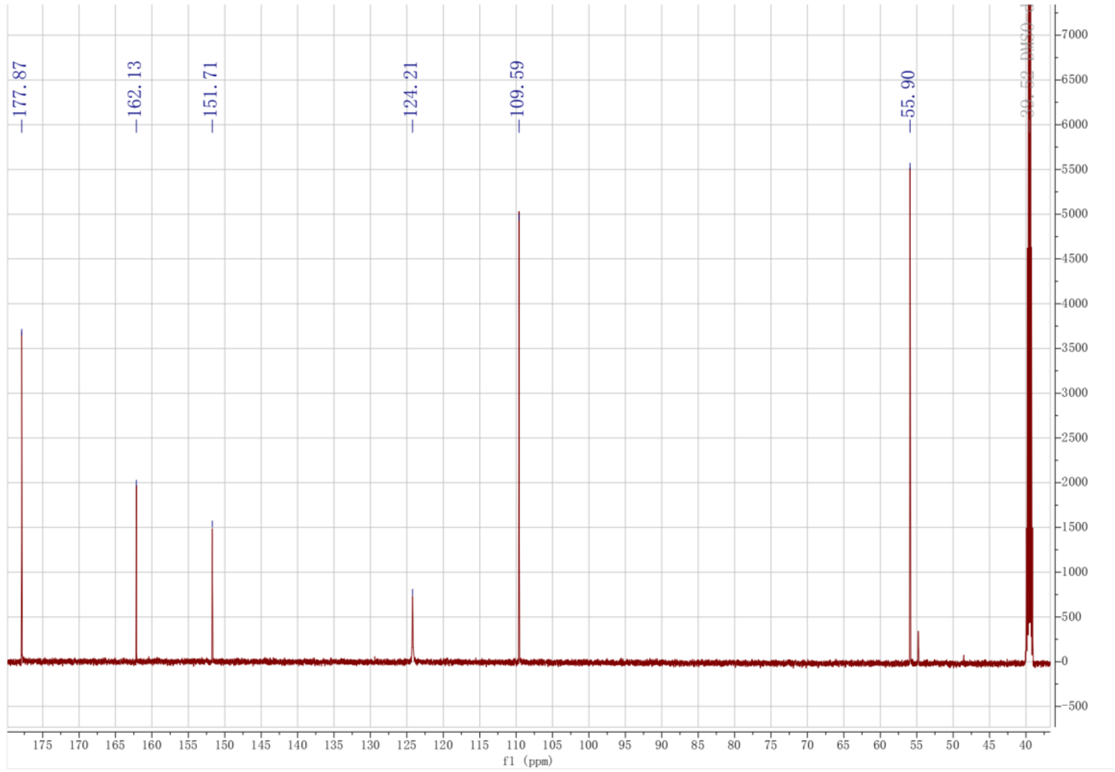

Fig. S40. ^{13}C-NMR spectra of compound **20** (151 MHz, DMSO-d_6)

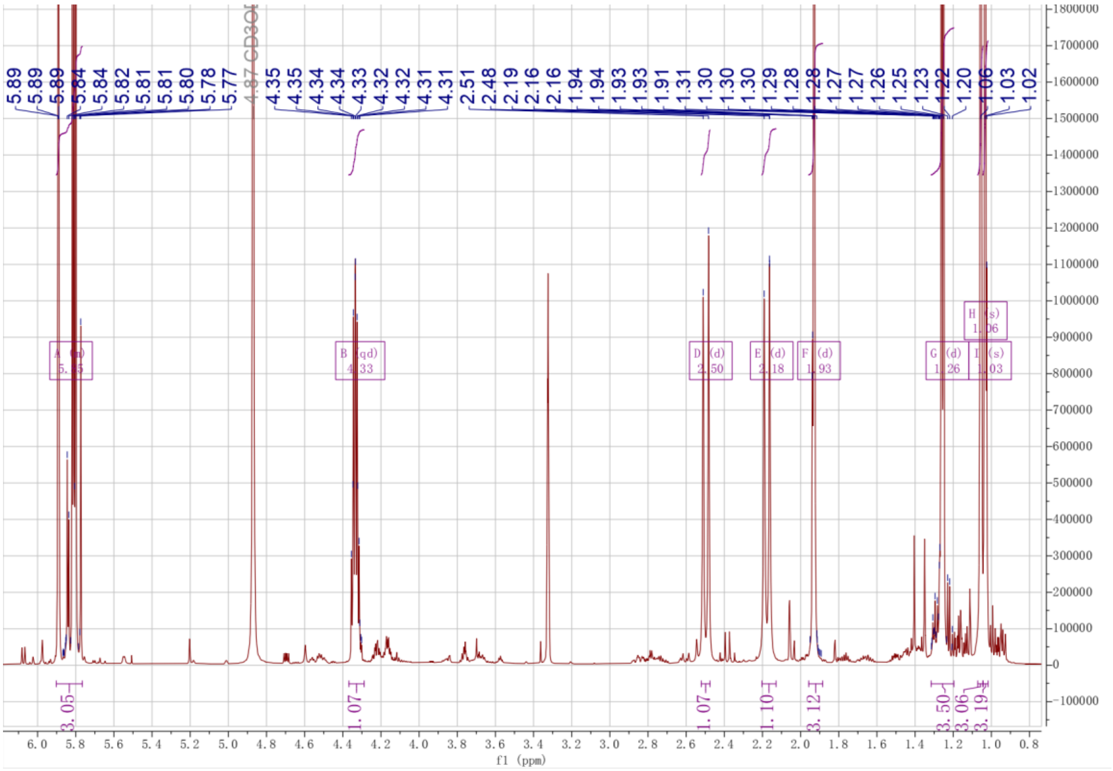

Fig. S41. ^{1}H-NMR spectrum of compound **21** (600 MHz, CD_3OD)

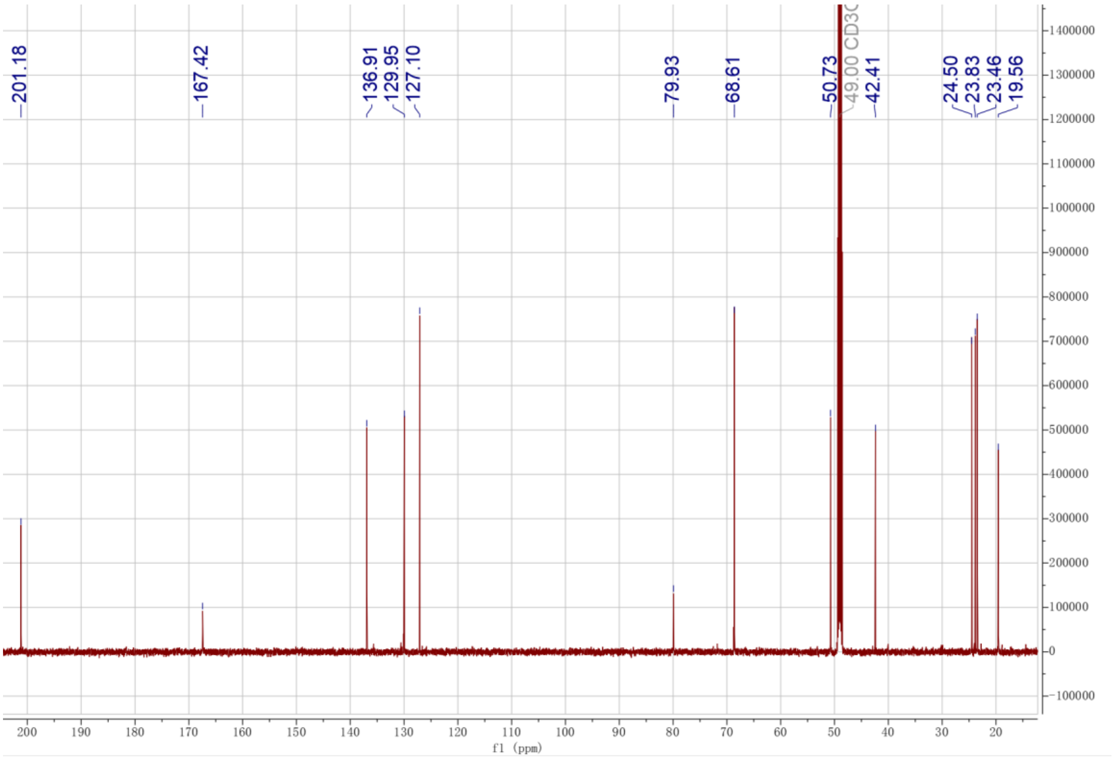

Fig. S42. ^{13}C-NMR spectra of compound **21** (151 MHz, CD_3OD)

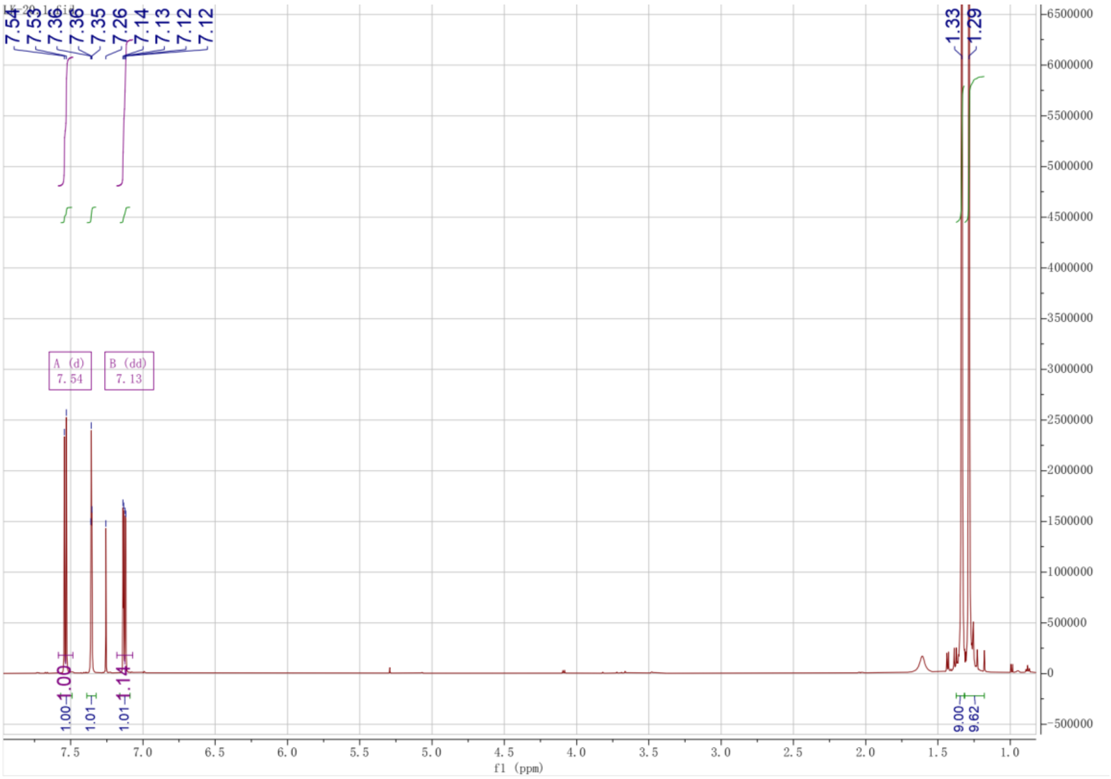

Fig. S43. ^{1}H-NMR spectrum of compound **22** (600 MHz, $CDCl_3$)

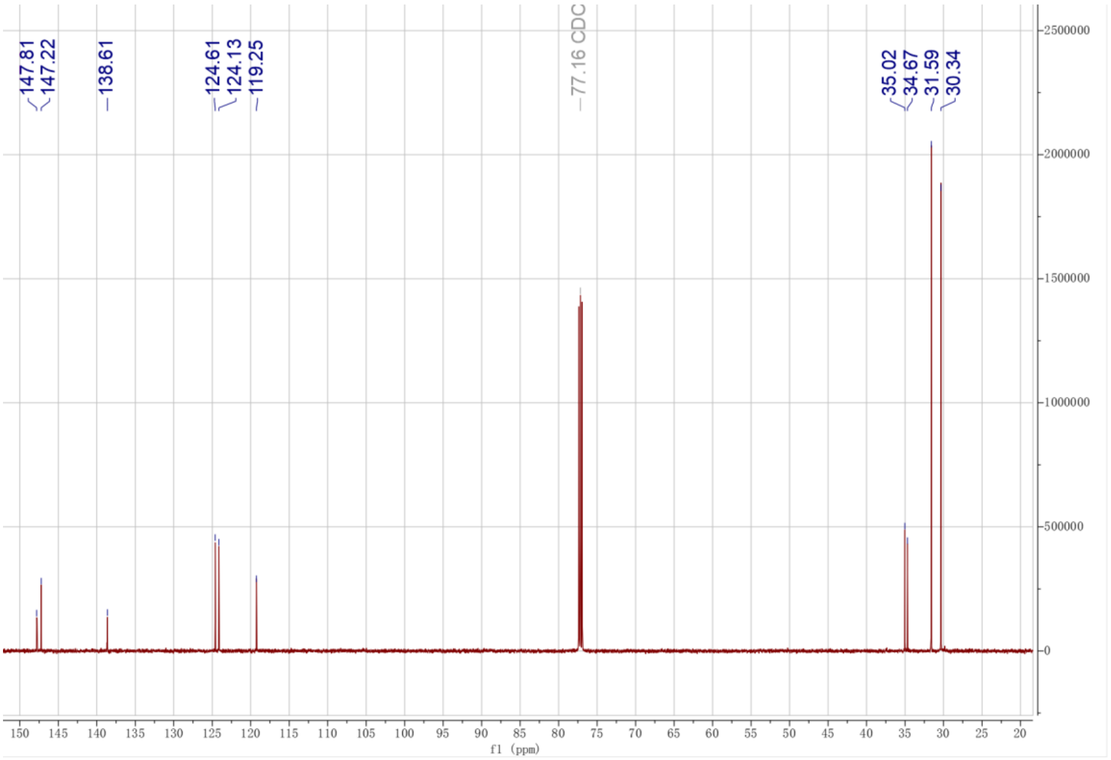

Fig. S44. ^{13}C-NMR spectra of compound **22** (151 MHz, $CDCl_3$)

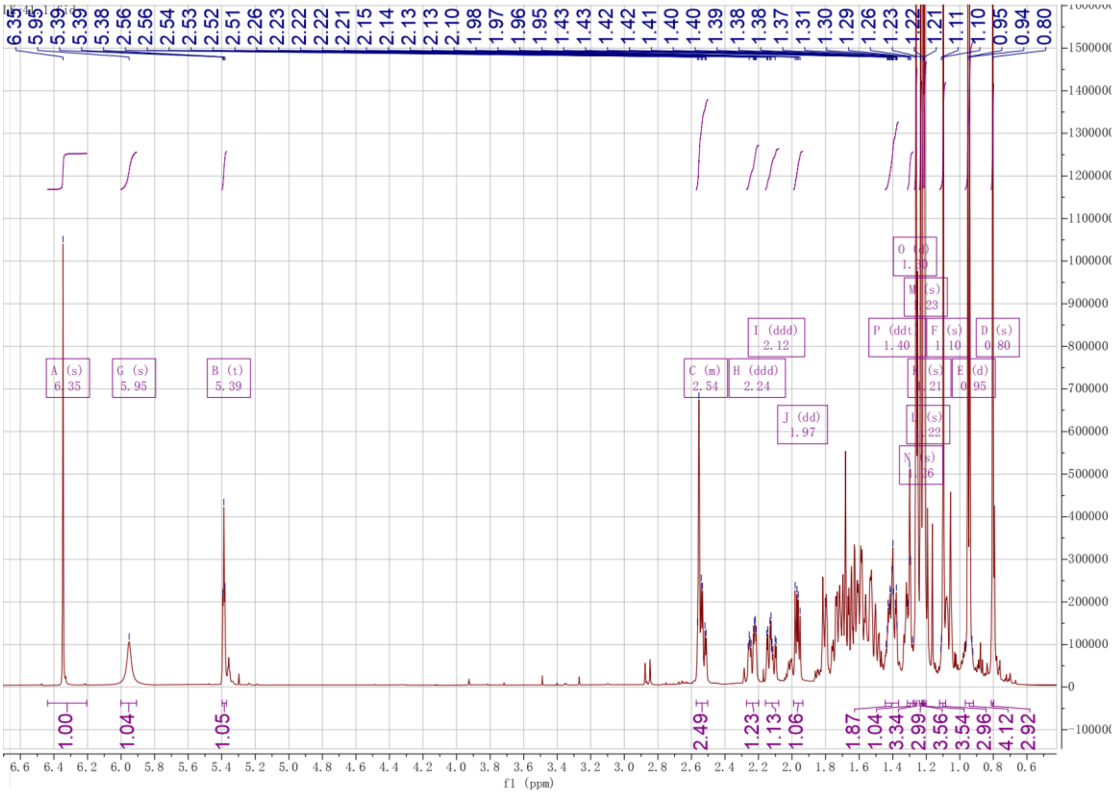

Fig. S45. ^{1}H-NMR spectrum of compound **23** (600 MHz, $CDCl_3$)

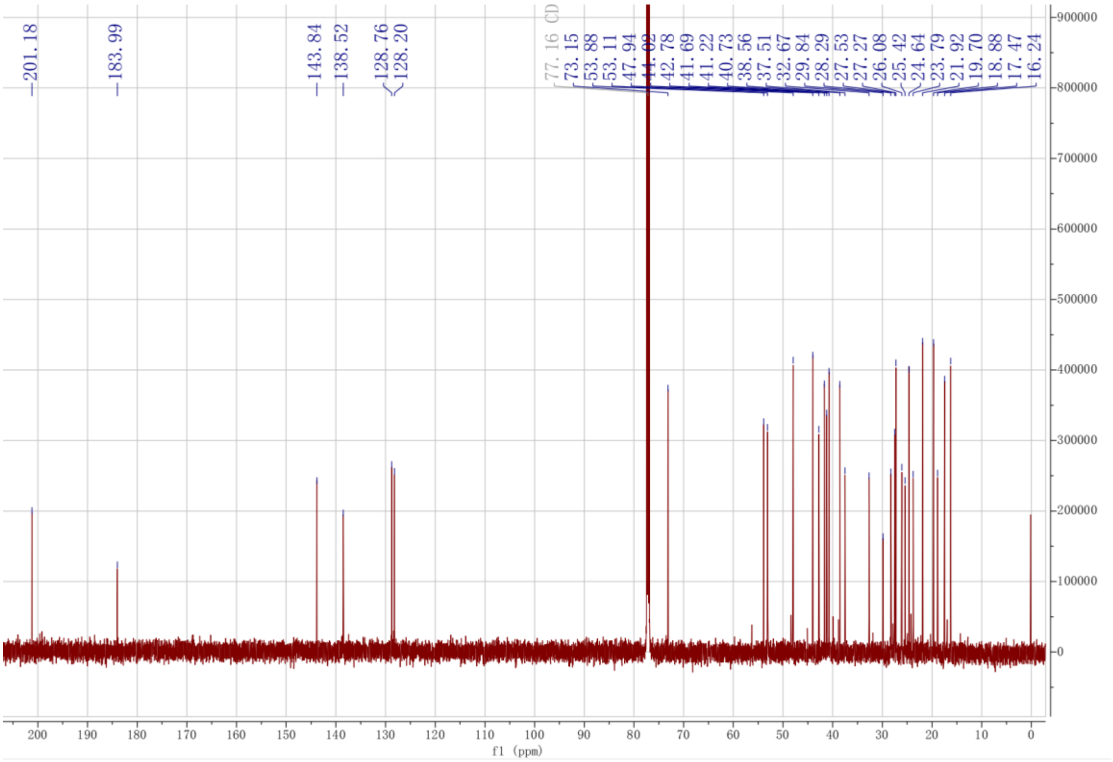

Fig. S46. ^{13}C-NMR spectra of compound **23** (151 MHz, $CDCl_3$)

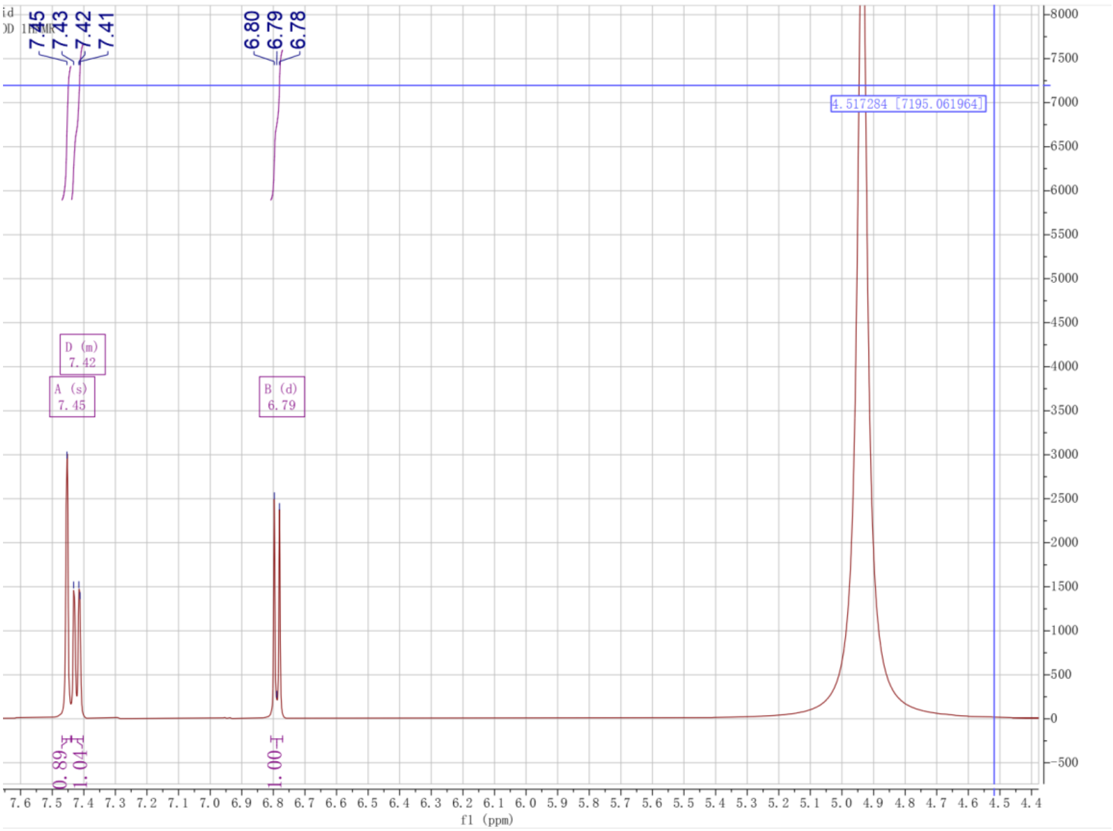

Fig. S47. ^{1}H-NMR spectrum of compound **24** (600 MHz, CD_3OD)

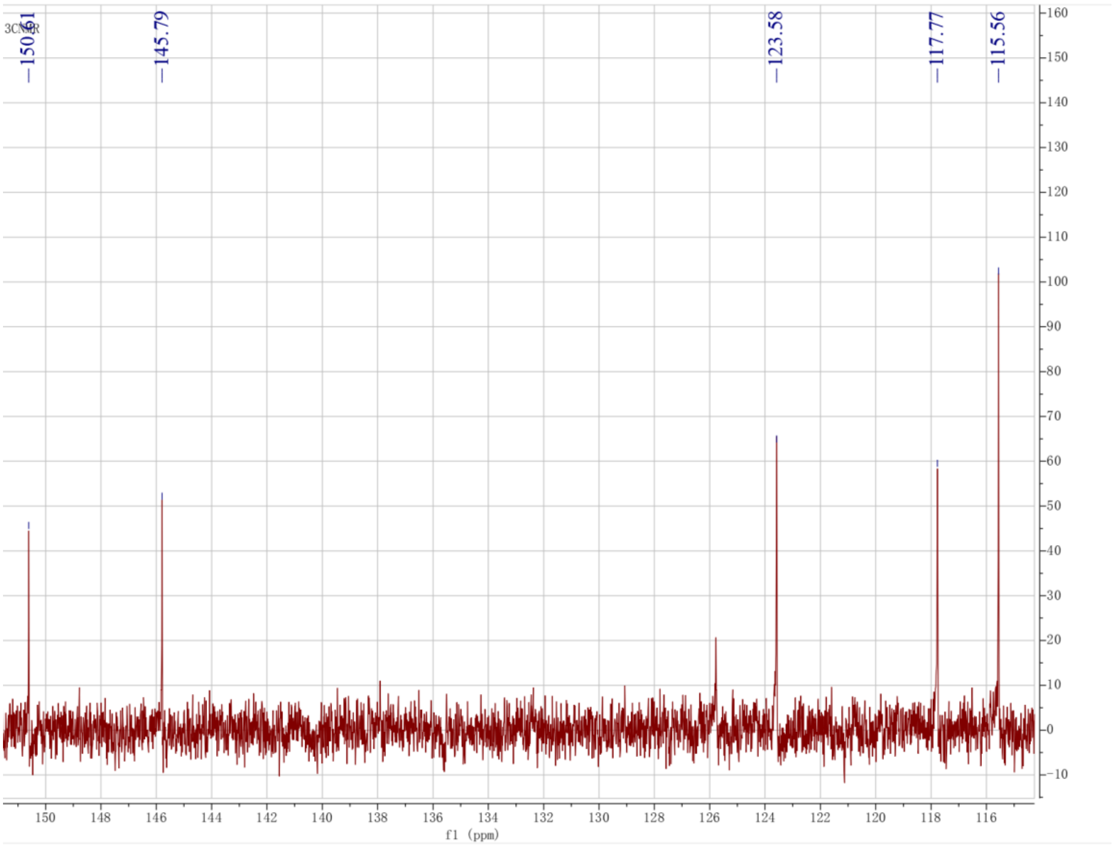

Fig. S48. ^{13}C-NMR spectra of compound **24** (151 MHz, CD_3OD)

^{1}H and ^{13}C NMR data of compounds **1**–**24**

methyl chlorogenate (**1**): yellow powder, $C_{17}H_{20}O_9$. ^{1}H-NMR (600 MHz, CD3OD) δ 7.51 (d, J = 16.2 Hz, H-8′), 7.03 (d, J = 1.8 Hz, H-2′), 6.93 (dd, J = 8.4, 1.8 Hz, H-6′), 6.76 (d, J = 8.4 Hz, H-5′), 6.19 (d, J = 16.2 Hz, H-7′), 5.25 (m, H-5), 4.11 (m, H-3), 3.71 (dd, J = 7.8, 3.0 Hz, H-4), 3.69 (s, 3′-OCH3), 2.22-2.09 (m, H-2, 6) , 2.04-1.98 (m, H-2a); ^{13}C-NMR (150 MHz, CD3OD) δ 175.4 (-COOH), 168.3 (C-9′), 149.7 (C-3′), 147.2 (C-8′), 146.9 (C-4′), 127.6 (C-1′), 123.0 (C-6′), 116.5 (C-5′), 115.1 (C-2′), 115.0 (C-7′), 75.8 (C-1), 72.5 (C-5), 72.1 (C-3), 70.3 (C-4), 52.9 (3′-OCH3), 38.0 (C-2, 6).

butyl chlorogenate (**2**): yellow powder, $C_{20}H_{26}O_9$. ^{1}H-NMR(CD_3OD,600 MHz) δ 7.52 (d, J = 15.9 Hz, H-7') , 7.04 (d , J = 1.2 Hz, H-2') , 6.95 (dd , J = 8.2, 2.1 Hz, H-6') , 6.78 (d , J = 7.8 Hz, H-5') ,6.21 (d , J = 15.6 Hz, H-6') , 5.26 (m, H-3) , 4.13 (m , H-5) , 4.05 (t , J = 6.8 Hz , H-8) , 3.73 (dd , J = 7.4 , 2.8 Hz , H-4), 2.18-2.12 (m, H-2e , H-6e), 2.22 (dd , J = 14.4 , 1.8Hz , H-6a) , 2.01 (dd , J = 13.2 , 7.2Hz , H-2a) , 1.61 (m , H-9) , 1.34 (m , H-10) , 0.96 (t , J = 7.4 Hz , H-11); ^{13}C-NMR (CD_3OD , 150MHz) δ 175.03 (C-7), 168.25 (C-9'), 149.70 (C-4'), 147.21 (C-7'), 146.87 (C-3'), 127.63 (C-1'), 122.97 (C-6'), 116.52 (C-5'), 115.11 (C-2'), 115.03 (C-8'), 75.73 (C-1), 72.45 (C-5), 72.18 (C-4), 70.21 (C-3), 66.36 (C-8), 38.02 (C-2), 37.64 (C-6), 31.62 (C-9), 20.08 (C-10), 14.00 (C-11).

ethyl chlorogenate (**3**): yellow powder, $C_{18}H_{22}O_9$. ^{1}H-NMR (CD_3OD , 600 MHz) : δ 7.52(d , J = 15.9 Hz , H-7'), 7.04 (m , H-2'), 6.94 (dd , J = 8.1 , 1.2 Hz , H-6'), 6.78 (d , J = 8.1 Hz , H 5'), 6.21 (d , J = 15.9 Hz , H-8'), 5.27 (m , H-3), 4.13 (m , -CH2 CH3), 4.13 (m , H-5), 3.74 (m , H-4), 1.97-2.24 (m , H-2), 1.97-2.24 (m , H-6), 1.24 (t , J = 7.1 Hz , -CH2 CH3) ; ^{13}C-NMR (CD_3OD , 150MHz) δ 174.9 (C-7), 168.3 (C-9'), 149.7 (C-4'), 147.1 (C-7'), 146.8 (C-3'), 127.6 (C-1'), 122.9 (C-6'), 116.5 (C-5'), 115.0 (C-2'), 115.0 (C-8'), 75.5 (C-1), 72.6 (C-4), 72.1 (C-3), 69.6 (C-5), 62.5 (-CH2 CH3), 38.0 (C-2), 36.2 (C 6), 14.3 (-CH2 CH3).

chlorogenic acid (**4**): yellow powder, $C_{16}H_{18}O_9$. ^{1}H-NMR (600 MHz, CD_3OD) δ: 7.55 (d, J = 15.9 Hz, H-7'), 7.04 (d, J = 2.1 Hz, H-2'), 6.95 (dd, J = 8.2, 2.1 Hz, H-6'), 6.77 (d, J = 8.2 Hz, H-5'), 6.26 (d, J = 15.9 Hz, H-8'), 5.33 (td, J = 9.2, 4.4 Hz, H-5),

4.16 (m, H-3), 3.72 (m, H-4), 2.02–2.25 (m, H-2, 6); ^{13}C-NMR (151 MHz, CD_3OD) δ: 177.00(C-7), 168.66 (C-9'), 149.56 (C 4'), 147.11 (C-3'), 146.80 (C-7'), 127.81 (C-1'), 123.04 (C-6'), 116.56 (C 5'), 115.26 (C-2'), 115.14 (C-8'), 76.13 (C-1), 73.48 (C-5), 71.94 (C-4), 71.36 (C-3), 38.77 (C-6), 38.21 (C-2).

cryptochlorogenic acid methyl ester (**5**): yellow powder, $C_{17}H_{20}O_9$. ^{1}H-NMR (600 MHz, CD_3OD) δ 7.64 (d, J = 15.9 Hz, H-8) 7.07 (d, J = 2.1 Hz, H-2′) 6.97 (dd, J = 8.2, 2.1 Hz, H-6′) 6.79 (d, J = 8.1 Hz, H-5′) 6.37 (d, J = 15.9 Hz, H-8) 4.83 (dd, J = 8.7, 3.1 Hz, H-4) 4.30 (m, H-3) 4.26 (m,H-5) 3.76 (s, 7-OCH3) 2.24-2.14 (m,H-2b, H-6b) 2.06 (1H, H-2b),2.02 (1H , m , H-6b); ^{13}C-NMR (151 MHz, CD_3OD) δ:175.66 (C-7), 168.94 (C-9′), 149.59 (C-4′), 147.14 (C-7′), 146.83 (C-3′), 127.85 (C-1′), 122.97 (C-6′), 116.48 (C-5′), 115.33 (C-8′), 115.15 (C-2′), 78.57 (C-4), 76.42 (C-1), 69.02 (C-3), 65.72 (C-5), 52.94 (7-OCH_3), 42.16 (C-2), 38.43 (C-6).

caffeic acid (**6**): white powder, $C_9H_8O_4$, ^{1}H-NMR (600 MHz, CD_3OD) δ 6.21 (d, J = 16.0 Hz, H-8), 6.77(d, J = 8.0 Hz, H-5), 6.93 (dd, J = 2.0, 8.0 Hz, H-6), 7.03 (d, J = 2.0 Hz, H-2), 7.52 (d, J = 16.0 Hz, H-7); ^{13}C-NMR (151 MHz, CD_3OD) δ: 171.02 (C-9), 149.44 (C-4), 147.00 (C-7), 146.78 (C-3), 127.81 (C-1), 122.90 (C-6), 116.56 (C-5), 115.49 (C-8), 115.04 (C-2).

p-coumaricacid (**7**): yellow powder, $C_9H_8O_3$, ^{1}H-NMR (600 MHz, DMSO-d6) δ 7.28 (d, J = 8.6 Hz, H-2,6), 7.11 (d, J = 15.8 Hz,H-7), 6.77 (d, J = 8.6 Hz, H-3, 5), 6.20 (d, J = 15.8 Hz, H-8); ^{13}C-NMR (151 MHz, DMSO-d6): δ 171.7 (C-9), 158.4 (C-4), 136.6 (C-7), 128.4 (C-2, 6), 126.9(C-1), 125.5 (C-3, 5), 115.7(C-8).

benzoic acid (**8**): white powder, $C_7H_6O_2$, ^{1}H-NMR (600 MHz, CD_3OD) δ 8.03 (m, J = 7.1, H-2,6), 7.46 (t, J = 7.8, H-3,5), 7.58 (t, J = 7.4, H-4), 13C-NMR (151 MHz, CD_3OD): 131.92 (C-1); 130.70 (C-2,6); 129.43 (C-3,5), 134.00 (C-4); 169.86 (C-7).

protocatechuic acid (**9**): white powder, $C_7H_6O_4$, ^{1}H-NMR (600 MHz, CD_3OD) δ 7.40 (d, J = 2.1 Hz, H-2), 7.38 (d, J = 2.0 Hz, H-6), 6.76 (d, J = 8.1 Hz, H-5). ^{13}C-NMR (151 MHz, CD_3OD) δ 170.23 (C=O), 151.49 (C-4), 146.02 (C-3), 123.91 (C-6), 123.19 (C-1), 117.77 (C-5), 115.78 (C-2).

ethylmethyl malate isomers (**10a/10b**): light yellow liquid, $C_7H_{12}O_5$, ^{1}H-NMR (600 MHz, $CDCl_3$) δ 4.27 (m, H-2,3′), 3.86-4.10 (m, H-6,6′), 3.42-3.61 (dd, H-5,5′), 2.52-2.65 (m, H-3,2′) 1.05 (m, H-7,7′); ^{13}C-NMR (151 MHz, $CDCl_3$): δ 173.4, 173.9 (C-4, 1′), 170.6, 171.0 (C-1, 4′) 67.5, 67.5 (C-2,3′) 61.1, 62.1 (C-6, 6′) 52.0, 52.8 (C-5, 5′) 38.7, 38.9 (C-3, 2′) 14.2(C-7, 7′).

butylmethyl malate isomers (**11a/11b**): light yellow liquid, $C_9H_{16}O_5$, ^{1}H-NMR (600 MHz, CDCl3) δ 4.48 (2H, m, H-2, 3′), 4.23-4.02 (4H, m, H-5, 5′), 3.80-3.67 (6H, m, H-9, 9′), 2.86-2.73 (4H, m, H-3, 2′), 1.60 (4H, m, H-6, 6′), 1.35 (4H, m, H-7, 7′), 0.91 (6H, m, H-8, 8′);^{13}C-NMR (151 MHz, CDCl3) δ 170.98, 170.68 (C-1, 4′), 67.47, 67.42 (C-2, 3′), 38.82, 38.73 (C-3, 2′), 173.86, 173.50 (C-4, 1′), 65.98, 65.01 (C-5, 5′), 30.67, 30.61 (C-6, 6′), 19.15, 18.09 (C-7, 7′), 13.71, 13.67 (C-8, 8′), 52.81, 52.01 (C-9, 9′).

bibutyl malate (**12**): light yellow liquid, $C_{12}H_{22}O_5$, ^{1}H-NMR (600 MHz, $CDCl_3$) δ 4.46 (t, J = 5.0 Hz, H-2), 4.18 (2H, m, H-5), 4.09 (t, J = 6.7Hz, H-5′), 2.82 (dd, J = 16.2, 4.1Hz, H-3a), 2.76 (dd, J = 16.0, 6.1Hz, H-3b), 1.67-1.55 (m, 6, 6′), 1.41-1.31 (m, 7, 7′), 0.97-0.87 (m, 8, 8′); ^{13}C-NMR (600 MHz, CDCl3) δ 170.63 (C-1), 67.46 (C-2), 38.90 (C-3), 173.56 (C-4), 64.97 (C-5), 30.69, 30.63 (C-6, 6′), 19.17, 19.12 (C-7, 7′), 13.73, 13.69 (C-8, 8′), 65.94 (C-5′).

butyl-2-hydroxysuccinate (**13**): light yellow liquid, $C_8H_{14}O_5$, ^{1}H-NMR (600 MHz, CD_3OD) δ 4.47 (dd, J = 7.1, 4.9Hz, H-2), 4.15 (m, J = 10.8, 6.6Hz, H-3), 2.78–2.63 (m, 2H, H-1′), 1.67-1.59 (m, H-2′), 1.39 (dt, J = 14.9, 7.5Hz, H-3′), 0.94 (t, J = 7.4Hz, H-4′).^{13}C-NMR (151 MHz, CD_3OD) δ 174.74 (C-1), 173.85 (C-4), 68.70 (C-2), 66.11 (C-1’), 39.92 (C-3), 31.69 (C-2′), 20.05 (C-3′), 13.93 (C-4′).

dimethylmalate (**14**): light yellow liquid, $C_6H_6O_3$, ^{1}H-NMR (600 MHz, $CDCl_3$) δ 4.50 (dd, J = 5.3 Hz, H-2), 3.81 (s, 1-OCH_3), 3.71 (s, 4-OCH_3), 3.22 (d, J = 5.5 Hz, -OH), 2.90-2.76 (m, H-3). ^{13}C-NMR (151 MHz, $CDCl_3$) δ 38.6 (C-3), 52.1 (4-OCH_3), 53.0 (1-OCH_3), 67.4 (C-2), 171.1 (C-1), 173.8 (C-4).

methylmalate (**15**): light yellow liquid, $C_5H_8O_5$, ^{1}H-NMR (600 MHz, CD_3OD) *δ*: 4.50 (dd, J = 7.3, 4.7 Hz, H-2) 3.74 (s, -OCH3) 2.71 (m, H-3); ^{13}C-NMR (151 MHz, CD_3OD) *δ* 175.15 (C-4), 173.91 (C-1), 68.61 (C-2), 52.64 (-OCH3), 39.87 (C-3).

oleanolic acid (**16**): white powder, $C_{30}H_{48}O_3$, ^{1}H-NMR ($CDCl_3$, 600 MHz) δ 5.28 (m, 1H), 3.22 (dd, J = 11.3, 4.2 Hz, 1H), 2.82 (m, 1H) 1.14 (s, 3H), 0.99 (s, 3H), 0.93 (s, 3H), 0.92 (s, 3H), 0.91 (s, 3H), 0.78 (s, 3H), 0.76 (s, 3H). ^{13}C-NMR ($CDCl_3$, 151 MHz) δ 182.9 (C-28), 143.7 (C-13), 122.7 (C-12), 79.1 (C-3), 55.3 (C-2), 47.7 (C-23), 46.6 (C-5), 46.0 (C-9), 41.7 (C-17), 41.1 (C-1), 39.4 (C-19), 38.9 (C-14), 38.5 (C-4), 37.2 (C-18), 33.9 (C-8), 33.2 (C-10), 32.7 (C-21), 32.5 (C-22), 30.8 (C-29), 28.2 (C-7), 27.8 (C-20), 27.3 (C-15), 26.0 (C-27), 23.7 (C-11), 23.5 (C-16), 23.0 (C-30), 18.4 (C-6), 17.2 (C-26), 15.6 (C-25), 15.4 (C-24).

ursolic acid (**17**): white powder, $C_{30}H_{48}O_3$, ^{1}H-NMR (600 MHz, DMSO-d_6) *δ*: 5.13 (1H, dd, J = 13.7; 3.5 Hz; H-12); 3.01 (1H, dd, J = 5.2; 9.5 Hz, H-3); 2.12 (1H, d; J = 11.1 Hz, H-18); 1.80 (2H, dd, J = 13.7; 3.5 Hz, H-11); 1.53 (2H, m, H-16); 1.59 (1H, s, H-9); 1.56 (2H, m, H-1); 1.54 (2H, m, H-22); 1.52 (1H, m, H-20); 1.47 (1H, m, H-6a); 1.43 (2H, m, H-2); 1.30 (1H, m, H-19); 1.29 (1H, m, H-6b); 1.29 (2H, m, H-21); 1.27 (2H, m, H-7); 1.05 (3H, s, H-27); 1.00 (2H, m, H-15); 0.92 (3H, d, J = 6.8 Hz, H-30), 0.90 (3H, s, H-23); 0.88 (3H, s, H-25); 0.82 (3H, d, J = 5.9 Hz, H-29); 0.76 (3H, s, H-26), 0.69 (3H, s, H-24), 0.67 (1H, s, H-5); ^{13}C-NMR (151 MHz, $CDCl_3$) *δ*: 178.25 (C-28), 138.18 (C-13), 124.68 (C-12), 76.89 (C-3), 54.79 (C-2), 52.43 (C-23), 47.00 (C-5), 46.81 (C-9), 41.63 (C-17), 38.44 (C-1), 38.40 (C-19), 38.37 (C-14), 38.27 (C-4), 38.17 (C-18), 36.52 (C-8), 36.30 (C-10), 32.69 (C-21), 30.17 (C-22), 28.30 (C-29), 28.19 (C-7), 26.98 (C-20), 23.82 (C-15), 23.32 (C-27), 23.19 (C-11), 22.84 (C-16), 17.99 (C-30), 16.97 (C-6), 16.90 (C-26), 16.06 (C-25), 15.25 (C-24).

β-sitosterol (**18**): white powder, $C_{29}H_{50}O$, ^{1}H-NMR (600 MHz, $CDCl_3$) *δ*: 0.70 (s, J=7.3 Hz, CH_3-29), 0.83 (m, J=6.5 Hz, CH_3-27), 0.86 (d, J=6.5 Hz, CH3-26), 0.91 (d, J=6.4 Hz, CH_3-21), 1.07 (s, CH_3-18), 1.11 (s, CH_3-19), 3.52 (m, H-3), 5.30 (m, H-1); ^{13}C-NMR (151 MHz, $CDCl_3$) *δ* 140.91 (C-5), 121.88 (C-6), 71.97 (C-3), 56.91 (C-14), 56.20 (C-17), 50.27 (C-9), 45.98 (C-24), 42.45 (C-13), 39.92 (C-4), 37.40 (C-12), 36.65

(C-20), 36.29 (C-10), 34.09 (C-22), 32.05 (C-8), 31.81 (C-2), 30.46 (C-7), 29.85 (C-25), 29.29 (C-1), 28.40 (C-16), 26.21 (C-23), 24.45 (C-15), 23.21 (C-28), 21.23 (C-11), 19.97 (C-27), 19.55 (C-21), 19.18 (C-26), 18.93 (C-19), 12.13 (C-29), 12.01 (C-18).

betulinic acid (**19**): white powder, $C_{29}H_{50}O$, ^{1}H-NMR(C_5D_5N, 600 MHz) δ 4.95 (1H, d, J = 1.64 Hz, Hα-30) , 4.80(1H, s, Hβ-30) , 3.53 (1H, m, H-3) , 1.79 (3H, s, H-29) , 1.22 (3H, s, H-27), 1.07 (3H, s, H-26), 1.06 (3H, s, H-23), 1.01 (3H, s, H-25), 0.82 (3H, s, H-24); ^{13}C-NMR (C_5D_5N, 151 MHz) δ 179.30 (C-28), 151.80 (C-20), 110.45 (C-30), 78.60 (C-3), 57.11 (C-17), 56.40 (C-5), 51.44 (C-9), 50.24 (C-18), 48.25 (C-9), 43.33 (C-14), 41.59 (C-8), 40.01 (C-4), 39.77 (C-1), 39.09 (C-13), 38.07 (C-10), 38.00 (C-22), 35.31 (C-7), 33.36 (C-16), 31.69 (C-15), 30.76 (C-21), 29.15 (C-23), 28.79 (C-2), 26.59 (C-12), 21.69 (C-11), 19.97 (C-29), 19.27 (C-6), 16.91 (C-25, 26), 16.83 (C-24), 15.39 (C-27).

5-hydroxymethylfurfural (**20**): brown crystal, $C_6H_6O_3$, ^{1}H-NMR (600 MHz, DMSO-d_6) δ 9.54 (s, 1H, -CHO), 7.48 (t, J = 2.8 Hz, 1H, H-3), 6.60 (d, J = 3.5 Hz, 1H, H-4), 5.56 (s, 1H, -OH), 4.50 (s, 2H, -CH2). ^{13}C-NMR (151 MHz, DMSO-d_6) δ 177.87 (-CHO), 162.13 (C-5), 151.71 (C-2), 124.21 (C-3), 109.59 (C-4), 55.90(-CH2).

vomifoliol (**21**): colourless liquid, $C_{13}H_{20}O_3$, ^{1}H-NMR (600 MHz, CD_3OD) δ 5.90-5.77 (m, 3H, H-4, 7, 8), 4.33 (qd, J = 6.4, 4.7 Hz, 1H, H-9), 2.50 (d, J = 16.9 Hz, 1H, H-2b), 2.18 (d, J = 16.9 Hz, 1H, H-2a), 1.93 (d, J = 1.4 Hz, 3H, H-11), 1.26 (d, J = 6.5 Hz, 3H, H-10), 1.06 (s, 3H, H-12), 1.03 (s, 3H, H-13). ^{13}C-NMR (151 MHz, CD_3OD) δ 201.18 (C-3), 167.42 (C-5), 136.91 (C-7), 129.95 (C-8), 127.10 (C-4), 79.93 (C-6), 68.61 (C-9), 50.73 (C-2), 42.41 (C-1), 24.50 (C-13), 23.83 (C-10), 23.46 (C-12), 19.56 (C-11).

2, 2′-oxybis (1, 4-di-tert-butylbenzen) (**22**): white powder, $C_{28}H_{46}O$, ^{1}H-NMR (600 MHz, $CDCl_3$) δ 7.54 (d, J = 8.6 Hz, 2H, H-6, 6′), 7.36 (t, J = 2.2 Hz, 2H, H-3, 3′), 7.13 (dd, J = 8.6, 2.5 Hz, 2H, H-5-5′), 1.33 (s, 18H, H-7, 7′-CH3), 1.29 (s, 18H, H-8, 8′-CH3). ^{13}C-NMR (151 MHz, $CDCl_3$) δ 30.3 (8, 8′-CH3), 31.6 (7, 7′-CH3), 34.67 (C-8, 8′), 35.0 (C-7, 7′), 119.3 (C-6, 6′), 124.1 (C-5, 5′), 124.6 (C-3, 3′), 138.6 (C-4, 4′), 147.2 (C-1, 1′), 147.8 (C-2, 2′).

fupenjic acid (**23**): white powder, $C_{30}H_{44}O_5$, ^{1}H-NMR(600 MHz,CDCl3) δ: 6.35 (1H, s, H-1), 5.97 (1H, s, -OH), 5.38(1H, t, H-12), 2.52 (1H, s, H-18), 2.22 (1H, m, H-11b), 2.13(1H, m, H-11a), 1.25 (3H, s, CH_3-27), 1.23 (3H, s, CH_3-25), 1.22 (3H, s, CH_3-23), 1.21 (3H, s, CH_3-29), 1.09 (3H, s, CH_3-24), 0.94 (3H, d, J = 6.6 Hz, CH_3-30), 0.80 (3H, s, CH_3-26) ; ^{13}C-NMR (151 MHz, $CDCl_3$): δ 201.18 (C-3), 183.99 (C-28), 143.84 (C-2), 138.52 (C-13), 128.76 (C-1), 128.20 (C-12), 73.15 (C-19), 53.88 (C-5), 53.11 (18), 47.94 (C-17), 44.02 (C-4), 42.78 (C-9), 41.69 (C-10), 41.22 (C-20), 40.73 (C-18), 38.56 (C-8), 37.51 (C-22), 32.67 (C-7), 28.29 (C-15), 27.53 (C-23), 27.27 (C-29), 26.08 (C-21), 25.42 (C-16), 24.64 (C-27), 23.79 (C-11), 21.92 (C-24), 19.70 (C-25), 18.88 (C-6), 17.47 (C-30), 16.24 (C-26).

1,2,4-benzenetriol (**24**), red crystal, $C_6H_6O_3$, ^{1}H-NMR (600 MHz, CD_3OD) δ: 7.45 (s, 1H), 7.42 (m, 1H), 6.79 (d, J = 8.2 Hz, 1H);^{13}C-NMR (150 MHz, CD_3OD) δ 150.61 (C-4), 145.79 (C-2), 125.78 (C-1), 123.58 (C-6), 117.77 (C-3), 115.56 (C-5).

Table S1: molecular docking parameters

Ligand	Receptor	Distance	Angle	Binding energy (kcal/ mol)
1	Glu802,<br>Met1038,<br>Gly799,<br>Gly1260,<br>Phe914	3.48 Å,<br>3.28 Å,<br>3.12 Å,<br>3.40 Å,<br>3.37 Å	150.9°<br>137.8°<br>175.6°<br>135.8°	-8.77
2	Thr1010,<br>Glu802,<br>Met1038,<br>Phe798,<br>Arg880,<br>Ala910,<br>Phe914	3.09 Å, 2.76 Å<br>3.41 Å,<br>3.31 Å,<br>2.90 Å,<br>3.34 Å,<br>3.30 Å,<br>3.63 Å	162.1°, 179,1°<br>157.0°<br>134.0°<br>147.4°<br>137.1°<br>152.4°	-7.76
3	Glu1261,<br>Glu802,<br>Gln767,<br>Phe914	3.33 Å,<br>3.43 Å, 3.24 Å<br>3.28 Å,<br>3.43 Å	158.3°<br>144.9°, 170.3°<br>134.5°	-8.67
4	Glu802,<br>Met1038,<br>Gly799,<br>Phe798,<br>Phe914	3.31 Å,<br>3.05 Å, 3.59 Å<br>3.07 Å,<br>3.31 Å,<br>3.30 Å	149.7°<br>134.6°, 148.7°<br>151.8°<br>132.7°	-8.45
5	Glu802,<br>Met1038,<br>Gly799,<br>Gly1260,<br>Phe914	3.35 Å,<br>3.89 Å,<br>3.39 Å,<br>3.48 Å,<br>3.30 Å	144.2°<br>141.4°<br>170.2°<br>120.3°	-9.21
6	Glu802,<br>Glu1261,<br>Gly799,<br>Phe914	3.23 Å,<br>2.71 Å.<br>3.17 Å,<br>3.39 Å	147.1°<br>156.7°<br>149.0°	-5.90
7	Glu802,<br>Glu1261,<br>Gly799,<br>Phe914	3.28 Å,<br>2.71 Å,<br>3.11 Å,<br>3.46 Å	142.3°<br>131.3°<br>165.6°	-5.68
8	Arg880,<br>Thr1010,<br>Phe914	3.13 Å, 3.37 Å<br>3.03 Å,<br>3.46 Å	130.1°, 146.4°<br>122.0°	-5.08
9	Glu802,<br>Thr1010,<br>Phe914	3.14 Å, 3.47 Å<br>2.88 Å,<br>3.61 Å	173.9°, 151.4°<br>164.7°	-5.44

AP	Glu802<br>Arg880<br>Thr1010<br>Phe914	3.21 Å,<br>3.11 Å, 3.34 Å<br>2.97 Å<br>3.68 Å,<br>3.88 Å	126.2°<br>139.9°,163.4°<br>124.5°	-4.18
